# The scaffold RhoGAP protein ARHGAP8/BPGAP1 synchronizes Rac and Rho signaling to facilitate cell migration

**DOI:** 10.1091/mbc.E21-03-0099

**Published:** 2023-02-21

**Authors:** Darren Chen Pei Wong, Catherine Qiurong Pan, Shi Yin Er, T. Thivakar, Tan Zi Yi Rachel, Sock Hong Seah, Pei Jou Chua, Tingting Jiang, Ti Weng Chew, Parthiv Kant Chaudhuri, Somsubhro Mukherjee, Agus Salim, Thike Aye Aye, Cheng Gee Koh, Chwee Teck Lim, Puay Hoon Tan, Boon Huat Bay, Anne J. Ridley, Boon Chuan Low

**Affiliations:** a Mechanobiology Institute, National University of Singapore, Singapore 117411; b Cell Signaling and Developmental Biology Laboratory, Department of Biological Sciences, National University of Singapore, Singapore 117558; c Department of Anatomy, Yong Loo Lin School of Medicine, National University Health System, Singapore 117594; d Melbourne School of Population and Global Health and School of Mathematics and Statistics, The University of Melbourne, Melbourne, Victoria 3052, Australia; e Department of Pathology, Singapore General Hospital, Singapore 169856; f Division of Molecular Genetics & Cell Biology, School of Biological Sciences, Nanyang Technological University, Singapore 637551; g Department of Biomedical Engineering, National University of Singapore, Singapore 117583; h School of Cellular and Molecular Medicine, University of Bristol, Bristol BS8 1TD, United Kingdom; i NUS College, National University of Singapore, Singapore 138593; University of Queensland

## Abstract

Rho GTPases regulate cell morphogenesis and motility under the tight control of guanine nucleotide exchange factors (GEFs) and GTPase-activating proteins (GAPs). However, the underlying mechanism(s) that coordinate their spatiotemporal activities, whether separately or together, remain unclear. We show that a prometastatic RhoGAP, ARHGAP8/BPGAP1, binds to inactive Rac1 and localizes to lamellipodia. BPGAP1 recruits the RacGEF Vav1 under epidermal growth factor (EGF) stimulation and activates Rac1, leading to polarized cell motility, spreading, invadopodium formation, and cell extravasation and promotes cancer cell migration. Importantly, BPGAP1 down-regulates local RhoA activity, which influences Rac1 binding to BPGAP1 and its subsequent activation by Vav1. Our results highlight the importance of BPGAP1 in recruiting Vav1 and Rac1 to promote Rac1 activation for cell motility. BPGAP1 also serves to control the timing of Rac1 activation with RhoA inactivation via its RhoGAP activity. BPGAP1, therefore, acts as a dual-function scaffold that recruits Vav1 to activate Rac1 while inactivating RhoA to synchronize both Rho and Rac signaling in cell motility. As epidermal growth factor receptor (EGFR), Vav1, RhoA, Rac1, and BPGAP1 are all associated with cancer metastasis, BPGAP1 could provide a crucial checkpoint for the EGFR-BPGAP1-Vav1-Rac1-RhoA signaling axis for cancer intervention.

## INTRODUCTION

Cancer metastasis is the major cause of cancer deaths (Chaffer and Weinberg, 2011; Hanahan, 2022). It comprises multiple steps of cell migration (Reymond *et al.*, 2013) that require active cytoskeleton remodeling, forming dynamic filopodia, lamellipodia, and invadopodia to allow cells to invade the surrounding tissues. Rho GTPases are key molecular switches that control actin dynamics in cell migration and are known to contribute to cancer progression (Ridley, 2015; Haga and Ridley, 2016). Mutations in Rho GTPases are frequent in only a few cancer types (Vega and Ridley, 2008). However, deregulation of Rho GTPase signaling is often associated with tumorigenesis at the level of gene expression or activation of Rho GTPases through their regulators or downstream effectors. Rho GTPases are activated by guanine nucleotide exchange factors (GEFs) and inactivated by GTPase-activating proteins (GAPs) (Haga and Ridley, 2016). The most well-characterized Rho GTPases are RhoA, Rac1, and Cdc42, which are known to mediate actomyosin contractility and lamellipodium and filopodium formation, respectively (Schmidt and Hall, 1998). Intriguingly, RhoA and Rac1 often act antagonistically and have distinct spatiotemporal activity profiles in the lamellipodia (Rottner *et al.*, 1999; Sander *et al.*, 1999; Machacek *et al.*, 2009). This is believed to involve active Rac1 or RhoA transducing signals to their downstream effectors that down-regulate RhoA or Rac1, respectively (Nimnual *et al.*, 2003; Ohta *et al.*, 2006; Wildenberg *et al.*, 2006). However, it remains unclear whether the distinct spatiotemporal activities of Rho and Rac can be regulated by a single protein entity that acts on both GTPases, facilitating a more efficient control in cell motility. We speculate that such an integrator for RhoA and Rac should bind to both RhoA and Rac1 to coregulate their activity in close proximity, at the right place and at the right time.

We previously identified a RhoGAP, BPGAP1 (also known as ARHGAP8), that acts as a GAP for RhoA and interacts with Rac1, but it is not a Rac1 GAP (Shang *et al.*, 2003). This raises the possibility that BPGAP1 could coregulate Rho and Rac1 activities. BPGAP1 is up-regulated in primary colorectal tumors (Johnstone *et al.*, 2004) and invasive cervical cancer (Song *et al.*, 2008). It is a multidomain RhoGAP and scaffolding protein that induces cell protrusions and cell migration via the interplay of its BCH domain, proline-rich region (PRR), and RhoGAP domain (Shang *et al.*, 2003). BPGAP1 also stimulates ERK activation (Lua and Low, 2005a; Pan *et al.*, 2010), leading to enhanced cancer cell proliferation (Jiang *et al.*, 2016) and cell motility (Pan *et al.*, 2010). Consistent with its prometastatic potential, the PRR of BPGAP1 interacts with the SH3 domain of the actin regulator cortactin and translocates cortactin to lamellipodia to enhance cell motility (Lua and Low, 2004). Therefore, we hypothesized that BPGAP1 could coordinate Rac1 and RhoA signaling in cell motility.

## RESULTS

### BPGAP1 expression is elevated in breast cancer and promotes breast cancer cell migration

**FIGURE 1:**
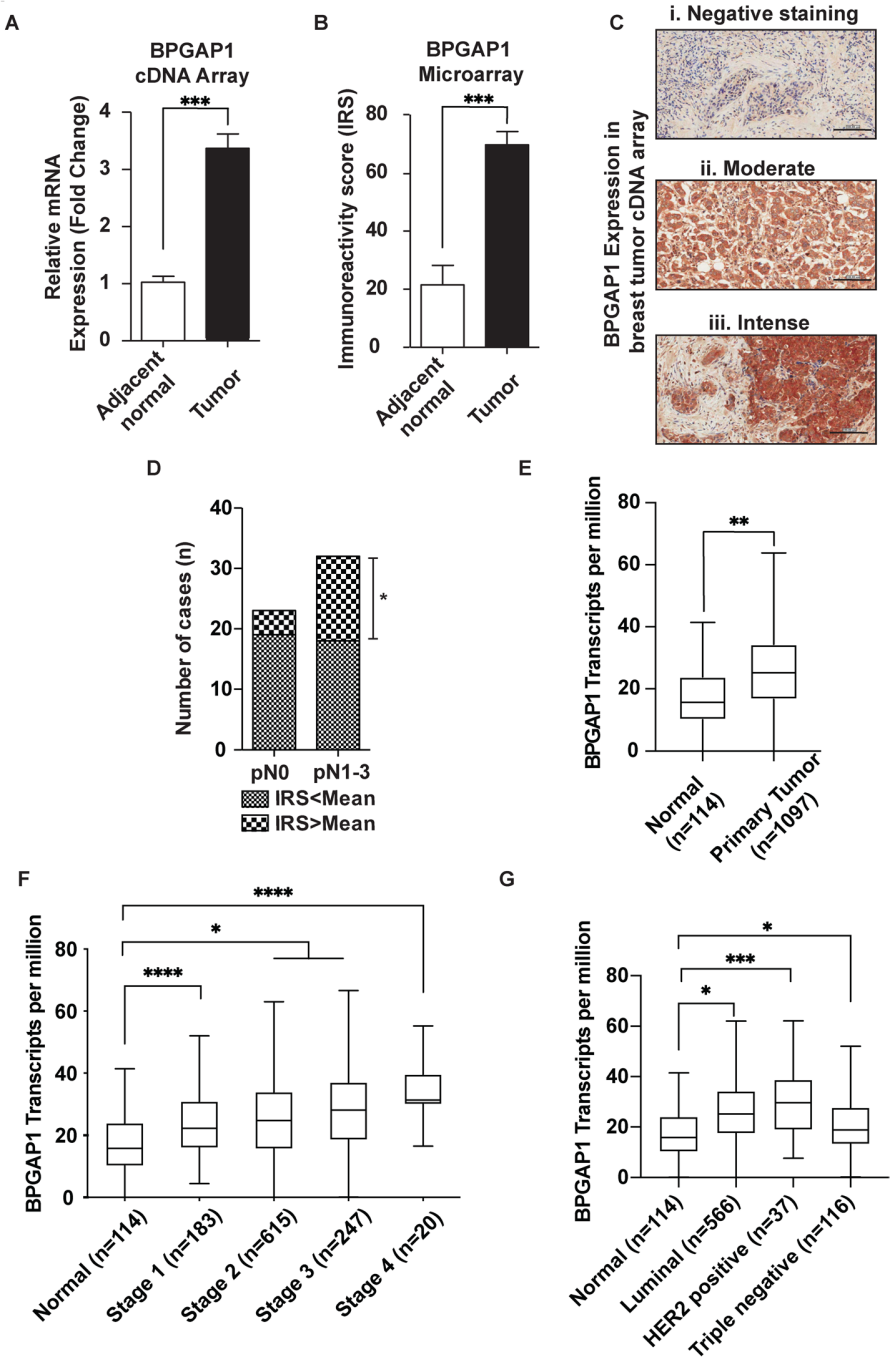
BPGAP1 expression is elevated in breast cancer. (A) mRNA expression of BPGAP1 is up-regulated in breast cancer. BPGAP1 expression was analyzed by real-time PCR from the cDNA of 48 breast cancer tissues. *** represents *P* < 0.001, nonparametric Mann–Whitney test. (B) BPGAP1 protein expression is up-regulated in breast cancer. BPGAP1 protein expression was analyzed by immunohistochemistry in 167 breast cancer samples. BPGAP1 was detected in the cytoplasm of breast tumor epithelial cells (C). The mean IRS of cytoplasmic staining for BPGAP1 is 70. Breast cancer cells showed statistically significant higher BPGAP1 expression with IRS mean score (69.66 ± 4.57, *n* = 167) compared with adjacent normal breast tissues (21.50 ± 6.71, *n* = 20). *** represents *P* < 0.001, nonparametric Mann–Whitney test. (D) BPGAP1 expression is associated with cancer cells metastasized to the lymph node. * represents *P* < 0.05, Chi-square test. (Please refer to Table 1 for clinical data.) (E) BPGAP1 is up-regulated in breast invasive carcinoma (BRCA). Through TCGA data, BPGAP1 expression is up-regulated in primary tumors. The Mann–Whitney *U* test was used, and ** indicates *P* < 0.01. (F) BPGAP1 expression increases with the stages of breast cancer. Relative expression of BPGAP1 in normal, Stage 1, Stage 2, Stage 3, and Stage 4 breast cancer from TCGA data. The box-and-whisker plots represent median and interquartile ranges, and one-way ANOVA was used to make multiple comparisons against the normal group. Gaussian distribution was assumed, and the Greisser–Greenhouse correction was used to account for sphericity differences. * indicates *P* < 0.05, and **** indicates *P* < 0.0001. (G) Expression of BPGAP1 is higher in luminal and HER2-positive BRCA patients*.* Relative expression of BPGAP1 in normal, luminal, HER2-positive, and triple-negative BRCA patients in TCGA data. The box-and-whisker plots represent median and interquartile ranges, and one-way ANOVA was used to make multiple comparisons against the normal group. Gaussian distribution was assumed, and the Greisser–Greenhouse correction was used to account for sphericity differences. * indicates *P* < 0.05, and *** indicates *P* < 0.001.

To examine the role of BPGAP1 in cancer development and its association with cancer metastasis, we first analyzed the expression profile of BPGAP1 in different tumors and normal tissues. Through GENT (Gene Expression Database of Normal and Tumor Tissue) analyses, BPGAP1 mRNA expression was abundant and highly up-regulated in breast cancer samples (Supplemental Figure S1). Consistently, higher levels of BPGAP1 expression were observed in breast tumor cDNA arrays (Figure 1A) and tissue microarrays (Figure 1B; with staining intensity in Figure 1C) as compared with normal breast tissues. The mean immunoreactivity score (IRS) of cytoplasmic staining for BPGAP1 is 70. Next, we used the mean IRS of BPGAP1 as the cutoff value to stratify BPGAP1 expression in breast cancer tissues into two groups for further clinicopathological analysis. The data showed that more patients with BPGAP1 staining greater than the mean IRS BPGAP1 (70) have cancer cells metastasized to lymph nodes (Figure 1D). To corroborate these observations, we further validated our findings with the available public cancer transcriptome database, UALCAN. Consistently, higher BPGAP1 expression was observed in primary breast tumors than in normal cells (Figure 1E) and in all stages of breast cancer as compared with control (Figure 1F). Furthermore, BPGAP1 expression was higher in luminal and HER2-positive (higher association with metastasis) breast cancer as compared with that in healthy subjects (Figure 1G).

To examine the role of BPGAP1 in breast cancer cells, we set out to generate stable breast cancer cell lines expressing BPGAP1. First, we assessed the BPGAP1 expression in two different breast cancer cell lines, MCF7 and MDA-MB-231. Interestingly, reverse transcription–PCR (RT-PCR) (Supplemental Figure S2A) and Western blot (Supplemental Figure S2B) showed that BPGAP1 was highly expressed in MCF7 compared with MDA-MB-231 cells, probably due to cancer cell heterogeneity and their different genomic mutation profiles. Nevertheless, our bioinformatic analysis (Figure 1 and Supplemental Figure S1) clearly associates higher BPGAP1 expression with the metastatic potential of breast cancer. To this end, we used MCF cells as the main model to elucidate the impact of BPGAP1 and its underlying mechanism of action in controlling cell migration and generated a stable MDA-MB-231 cell line that expressed either mCherry-BPGAP1 or mCherry vector control for their comparison.

**FIGURE 2:**
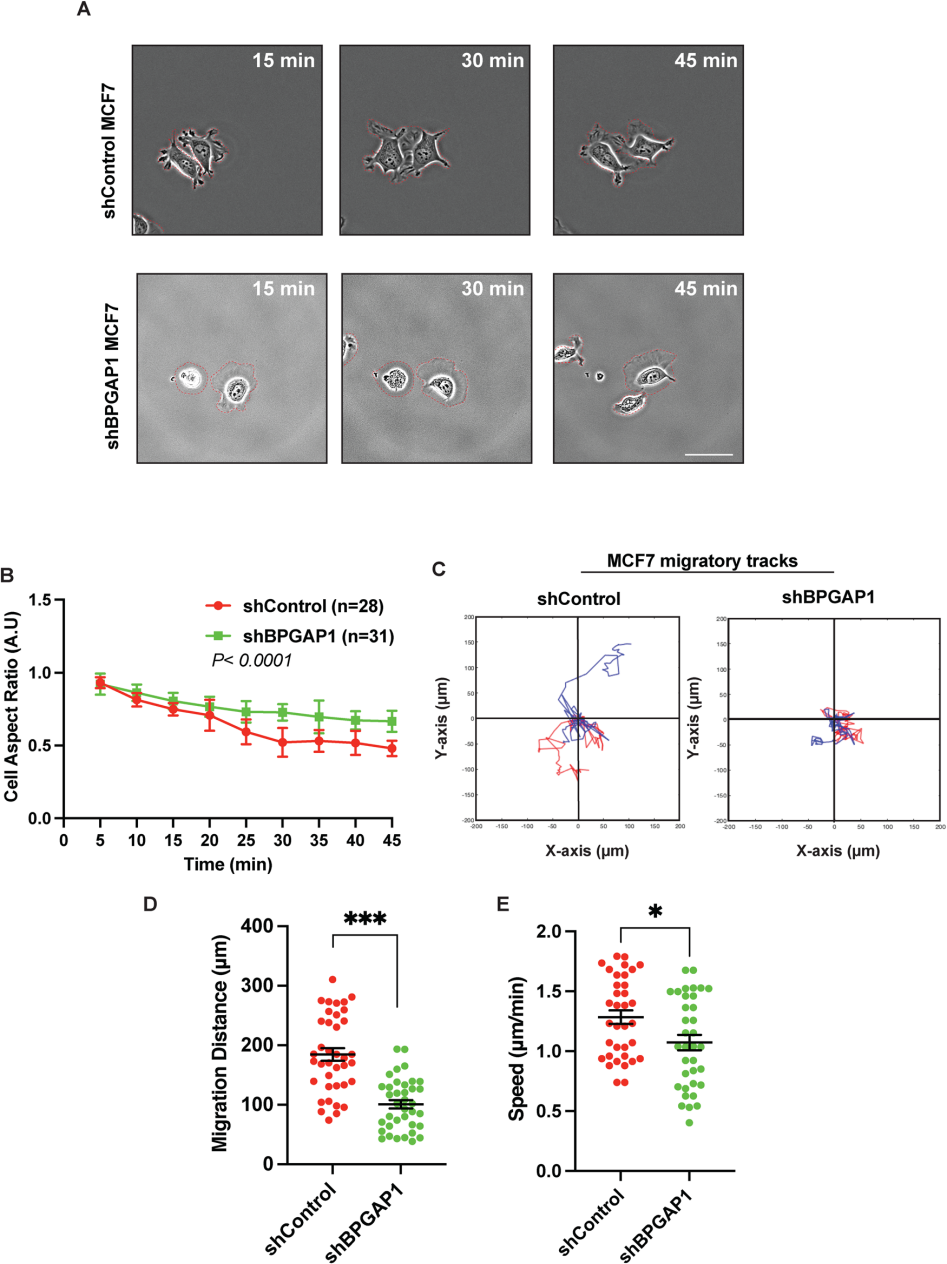
BPGAP1 is required for MCF7 breast cancer cell motility. (A) BPGAP1 promotes cell polarization and motility*.* MCF7 cells transfected with short hairpin RNA (shRNA) control or shRNA of BPGAP1 were seeded on collagen-coated glass bottom dishes. Time-lapse images of cells were acquired for 45 min. Representative images are shown. Scale bar: 30 μm. Red dashed lines demarcate the cell boundary. (B) Cell aspect ratio, (C) migration tracks (two representative tracks that were tracked over 2 h are illustrated in blue and red), (D) total distance migrated (over 2 h), and (E) speed of migration (tracked over 2 h) were quantified using ImageJ illustrated with in-house Matlab code. All the data above were obtained from three independent experiments and are represent as mean ± SEM. A two-tailed unpaired Student’s *T* test was used. *** represents *P* < 0.001, and * represents *P* < 0.05.

To investigate how BPGAP1 could affect cellular behavior that contributes to motility, we first examined its effect on breast cancer cell morphology during cell spreading (Supplemental Movies S1 and S2). During initial cell spreading, known as P1 (phase 1), cells have been reported to spread out in an isotropic manner with high Rac1 activity. Subsequently, they transit to a contractile phase (P2; phase 2) with high Rho activity and low Rac1 activity before cell polarization and migration (Gauthier *et al.*, 2012). MCF7 BPGAP1 knockdown cells were strikingly less polarized with lesser long and thin protrusions at the protrusion tips (Figure 2A) and had a higher aspect ratio (Figure 2B). To confirm the requirement of BPGAP1 for cell spreading, we corroborated these observations using MDA-MB-231 mCherry-BPGAP1–overexpressing cells (Supplemental Movies S3). Indeed, overexpression of mCherry-BPGAP1 significantly promoted the spreading and polarization of MDA-MB-231 cells during spreading (Supplemental Figure S3, A and B). Next, we investigated whether BPGAP1 altered cell migration. We tracked the movement of the cells through the displacement of the cell nucleus over time. As expected, BPGAP1 knockdown in MCF7 and BPGAP1 overexpression in MDA-MB-231 cells reduced and enhanced the migration distance and speed of the cells, respectively (Figure 2, C–E, and Supplemental Figure S3, C–E).

**Figure:** Movie S1 MCF7 cells transiently transfected with sh-vector control and seeded on collagen-coated dishes. Representative 50 minutes time-lapse movies of cells. Scale bar: 50 μm.

**Figure:** Movie S2 MCF7 cells transiently transfected with sh-BPGAP1 were seeded on collagen-coated dishes. Representative 50 minutes time-lapse movies of cells. Scale bar: 50 μm.

**Figure:** Movie S3 MDA-MB-231 cells stably expressing mCherry vector control (left) or mCherry-BPGAP1 (right) were seeded on collagen-coated dishes. Time-lapse movies of cells were acquired for 105 minutes; 3 frames per second. Scale bar: 70 μm.

Collectively, we show that high BPGAP1 transcript and protein expression are associated with breast cancer cells, particularly higher in late-stage and metastatic breast cancer cells. Along with our functional data, these results support the notion that BPGAP1 promotes cell polarization associated with lamellipodia and stimulates cell migration.

### BPGAP1 localizes to lamellipodia, interacts with inactive Rac1, and promotes Rac1 activation

**FIGURE 3:**
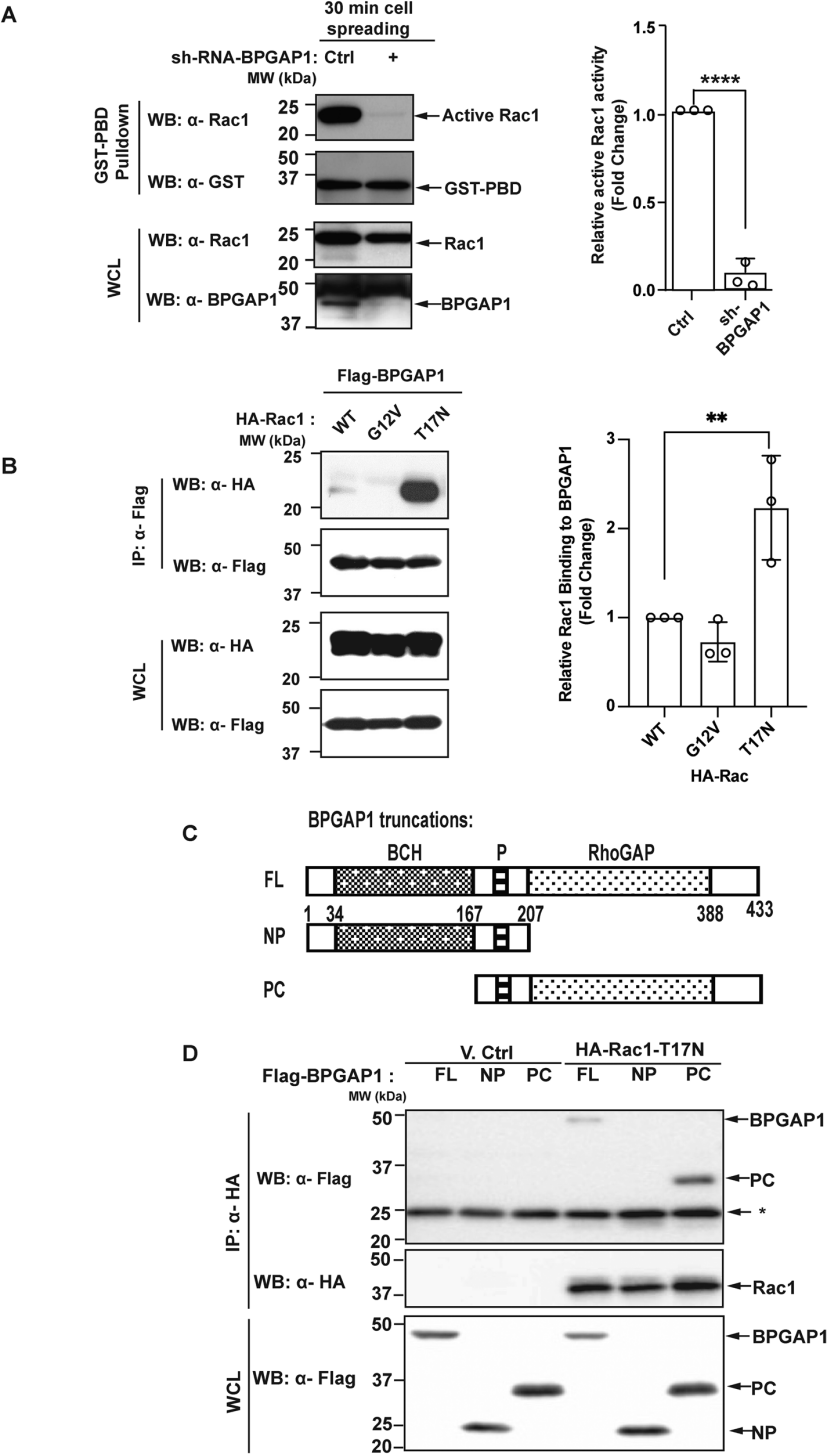
BPGAP1 interacts with inactive Rac1 and promotes Rac1 activation. (A) BPGAP1 is required for Rac1 activation. MCF7 cells transfected with shRNA control or shRNA of BPGAP1 were spread on collagen-coated wells for 30 min. Cells were lysed and incubated with GST-PBD beads that captured active Rac. Active Rac1 and total lysates were analyzed by immunoblotting. Quantification on the right represents mean ± SEM of three independent experiments. A two-tailed Student’s *T* test was used, and **** represents *P* < 0.0001. (B) BPGAP1 interacts with dominant negative form of Rac1. HEK293T cells were cotransfected with Flag-tagged BPGAP1 and wild-type (WT), constitutive active (G12V), or dominant negative (T17N) forms of Rac1. The cell lysates were incubated with anti-Flag M2 beads. Bound HA-tagged proteins and their expression in WCL were detected with anti-HA, while the precipitated Flag-tagged proteins were detected with anti-Flag antibodies. Quantification on the right represents mean ± SEM of three independent experiments. One-way ANOVA was used to compare the different conditions to the control group. ** represents *P* < 0.01. (C) Schematic diagram of BPGAP1 truncation mutants: full-length (FL), N-terminus with proline-rich (NP), and proline-rich and C-terminus (PC). (D) BPGAP1 interacts with Rac1 via the region containing the RhoGAP domain. HEK293T cells coexpressing the indicated constructs were lysed and immunoprecipitated with anti-HA beads. Bound and WCL proteins were analyzed by immunoblotting. * indicates antibody light chains.

BPGAP1 colocalizes in lamellipodia with cortactin (Lua and Low, 2004), which is known to regulate Arp2/3 complex–stimulated actin branching in lamellipodia, including in breast cancer MCF7 cells (Kirkbride *et al.*, 2011). Rac1 is a potent inducer of lamellipodia (Schmidt and Hall, 1998), and BPGAP1 is known to interact with Rac1 (Shang *et al.*, 2003). We, therefore, investigated whether BPGAP1 could regulate Rac1. Because BPGAP1 induces lamellipodia and protrusions at 30 min of cell spreading (Figure 2A and Supplemental Figure S3A), we determined the levels of active Rac1 after 30 min of cell spreading in MCF7 cells depleted of BPGAP1. Intriguingly, BPGAP1-depleted MCF7 cells had lower Rac1 activity after 30 min compared with that in the control cells (Figure 3A), implying that BPGAP1 is required for Rac1 activation to stimulate cell spreading and possibly cell motility.

To probe how BPGAP1 could increase Rac1 activity, we examined the nature of the interaction between BPGAP1 and Rac1. BPGAP1 preferentially interacted wit h the dominant negative form of Rac1, T17N (Figure 3B). It interacted weakly with the wild-type and no binding was detectable for the constitutively active Rac1, G12V (Figure 3B). Next, using BPGAP1 truncation mutants (Figure 3C), we showed that Rac1 associated with the C-terminal region (PC) of BPGAP1 that contains the RhoGAP domain but not with the N-terminal region (NP) that includes the BCH domain (Figure 3D).

Hence, our data show that BPGAP1 activation of Rac1 during cell spreading is associated with preferential binding of T17N dominant negative Rac1 to the RhoGAP domain of BPGAP1. Because BPGAP1 induces Rac1 activation and selectively interacts with the inactive form of Rac1 and yet is not a RacGEF, this raises the possibility that BPGAP1 could act as a scaffold protein that recruits a RacGEF for the activation of Rac1.

### BPGAP1 interacts with Vav1 upon EGF stimulation and during cell spreading

**FIGURE 4:**
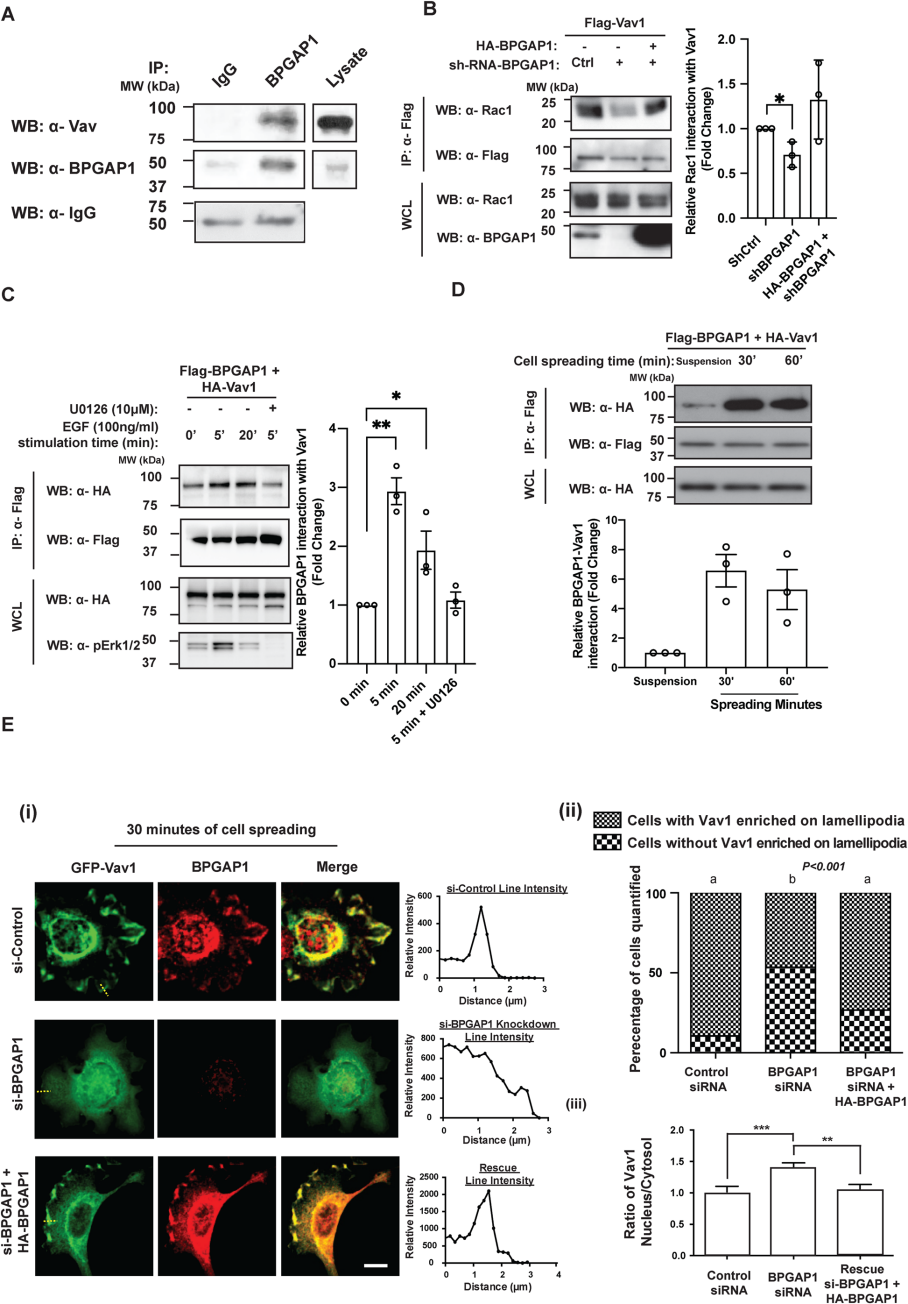
BPGAP1 interacts with Vav1 upon EGF stimulation and during cell spreading. (A) Endogenous Vav interacts with BPGAP1*.* MCF7 cells were lysed and incubated with IgG control or anti-BPGAP1 for 6 h before adding beads conjugated with protein A. Both bound and WCL proteins were analyzed by immunoblotting with the indicated antibodies, *n* = 3. (B) BPGAP1 promotes interaction of Vav1 and Rac1. MCF7 cells were transfected with Flag-tagged Vav1 with or without HA-tagged BPGAP1 in control or BPGAP1 knockdown cells. Bound proteins and lysate proteins were analyzed by immunoblotting. Quantification on the right represents mean ± SEM of three independent experiments. One-way ANOVA was used to compare the different conditions to the control group. * represents *P* < 0.05. (C) Interaction of BPGAP1 and Vav1 is enhanced upon EGF stimulation of Ras/MEK/ERK. MCF7 cells were transfected with Flag-BPGAP1 and HA-Vav1 and stimulated with 100 ng/ml EGF for the times indicated. The cells were treated in the absence or presence of MEK inhibitor, U0126 (10 μM), for 1 h before EGF stimulation. Cells were lysed and immunoprecipitated with anti-Flag beads. Both bound and WCL proteins were analyzed by immunoblotting. Quantification on the right represents mean ± SEM of three independent experiments. One-way ANOVA was used to compare the different conditions to the control group. * represents *P* < 0.05, and ** represents *P* < 0.01. (D) Interaction of BPGAP1 and Vav1 is enhanced upon cell spreading. MCF7 cells expressing Flag-tagged BPGAP1 and HA-tagged Vav1 were seeded onto collagen-coated plates and lysed after the indicated times. Cell lysates were immunoprecipitated with anti-Flag antibodies. Both immunoprecipitated and total lysate proteins were analyzed by immunoblotting. Quantification on the right represents mean ± SEM of three independent experiments. One-way ANOVA was used to compare the different conditions to the control group. * represents *P* < 0.05. (E) Vav1 is localized to lamellipodia in the presence of BPGAP1. (i) MCF7 cells transfected with GFP-Vav1 in control, BPGAP1 knockdown cells, or BPGAP1 knockdown cells with reconstitution of HA-BPGAP1. Cells were seeded on collagen-coated glass coverslips for 30 min, fixed, permeabilized, and immunostained with BPGAP1 antibody, followed by secondary antibody conjugated with Alexa Fluor 555. Images were acquired using confocal microscopy. Line profiles of Vav1 from lamellipodia to lamella (as represented by the yellow dotted lines) were analyzed using ImageJ. At least 50 cells were quantified. Scale bar: 10 μm. (ii) Data sharing different letters are statistically significant at *P* < 0.001, Chi-square. (iii) The cytosol/nucleus ratios of Vav1 from experiments in E(i) and Supplemental Figure S4D were quantified with ImageJ as described in *Materials and Methods*. Scale bars: 10 μm. All data are represented as mean ± SEM of three independent experiments. A two-tailed Student’s *T* test was used. ** represents *P* < 0.01, and *** represents *P* < 0.001.

Similar to BPGAP1, the Vav RacGEFs (Vav1, Vav2, and Vav3) have been reported to regulate adhesion-induced cell spreading, cell morphology, and migration (Gakidis *et al.*, 2004; Bhavsar *et al.*, 2009). These observations suggest that Vav proteins are potential candidates recruited by BPGAP1 for the activation of Rac1. Indeed, endogenous BPGAP1 coimmunoprecipitated with endo­genous Vav, supporting their physiological interaction in the cells (Figure 4A).

Next, we assessed the binding profile of BPGAP1 with Vav proteins. Through coimmunoprecipitation assays, BPGAP1 displayed similar binding profiles with Vav1, Vav2, and Vav3 (Supplemental Figure S4A). In normal tissues, Vav1 was reported to be hematopoietic cell–specific (Katzav *et al.*, 1989; Bustelo *et al.*, 1993), while Vav2 and Vav3 are expressed in multiple tissues. Interestingly, Vav1 was originally isolated as an in vitro–activated oncogene (Katzav *et al.*, 1989) and has been implicated in breast cancer (Du *et al.*, 2014), pancreatic adenocarcinoma, melanoma, and lung cancer (Fernandez-Zapico *et al.*, 2005; Bartolome *et al.*, 2006; Lazer *et al.*, 2009). Although it has been reported that MCF7 cells lack expression of Vav1 and that its overexpression stimulates apoptosis (Sebban *et al.*, 2013), we analyzed the online database and confirmed the expression of all Vav isoforms in MCF7 cells (Supplemental Figure S4B). We next verified this with RT-PCR analysis of total RNA from MCF7 and MDA-MB-231 cells and showed that Vav1, Vav2, and Vav3 were expressed (Supplemental Figure S4C). Several studies also confirmed the presence of Vav proteins in MCF7 and MDA-MB-231 cells (Grassilli *et al.*, 2014; He *et al.*, 2014; Chen *et al.*, 2015). These results reveal a possible context-dependent regulation of Vav1 in breast cancer cells dependent on the presence of BPGAP1. To verify this, we tested whether BPGAP1 was required for the interaction of Vav1 with Rac1. The coimmunoprecipitation of endogenous Rac1 with Flag-Vav1 was markedly reduced in BPGAP1-depleted MCF7 cells. However, their association was restored by the reintroduction of exogenous BPGAP1 in BPGAP1-depleted MCF7 cells, suggesting that BPGAP1 acts as a scaffold for Vav1/Rac1 interaction (Figure 4B).

To determine whether a physiological stimulus regulates BPGAP1 binding to Vav1, MCF7 cells were treated with EGF. EGF signaling was previously described to positively regulate cell spreading and migration in both two-dimensional (2D) and 3D cultures (Kim *et al.*, 2015; Ohnishi *et al.*, 2017). The association of BPGAP1 and Vav1 increased upon EGF stimulation (Figure 4C). BPGAP1 and Vav1 are both known to be involved in ERK/MAPK signaling (Villalba *et al.*, 2000; Lua and Low, 2005a). The EGF-induced increase in BPGAP1 interaction with Vav1 was prevented by treatment with the MEK inhibitor U0126 (Figure 4C), indicating that EGF acts via MEK/ERK to stimulate the interaction of BPGAP1 with Vav1.

Because both BPGAP1 (Figure 2 and Supplemental Figure S3) and Vav proteins (Gakidis *et al.*, 2004; Bhavsar *et al.*, 2009) regulate cell spreading, we investigated whether functions of BPGAP1 and Vav1 are coupled during cell spreading, viz., an increased association during cell spreading. As expected, BPGAP1 interaction with Vav1 increased as MCF7 cells attached to and spread on collagen-coated culture dish (Figure 4D). Furthermore, BPGAP1 and Vav1 colocalized at lamellipodia of MCF7 cells after 30 min of cell spreading (Figure 4E, i and ii). In strong contrast, Vav1 no longer localized to the cell edge in BPGAP1-depleted cells and this was restored by reintroducing BPGAP1 (Figure 4E, i and ii). Under steady-state conditions (24 h posttransfection), Vav1 localization was also affected by BPGAP1. Vav1 localized partly to the nucleus in BPGAP1-knockdown cells, whereas reintroducing BPGAP1 relocalized Vav1 from the nucleus to the cytosol (Figure 4Eiii and Supplemental Figure S4D). Taken together, these results suggest that BPGAP1 recruits the Vav1 that exited from the nucleus (albeit with an unknown mechanism) and/or cytoplasm to activate Rac1, thereby promoting lamellipodium formation.

To understand how BPGAP1 could promote Vav1 and Rac1 interaction, we examined which region of BPGAP1 interacted with Vav1. BPGAP1 has several proline-rich motifs that can be recognized by SH3 domains (Shang *et al.*, 2003; Lua and Low, 2004) (Supplemental Figure S5A), and Vav1 contains two SH3 domains (Bustelo, 2014). On the other hand, we have previously shown that the RhoGEF Lbc interacts with the BCH domain of another protein, BNIP-XL, via its DH-PH domain (Soh and Low, 2008). Indeed, Vav1 interacted with both the N-terminal and C-terminal regions of BPGAP1, with a higher association with the N-terminal region (Supplemental Figure S5B). This result indicates that BPGAP1 interacts with Vav1 preferentially via its BCH domain, whereas BPGAP1 interacts with inactive Rac1 via the C-terminal GAP domain (Figure 3D). We then determined the binding profile of BPGAP1 with wild-type Vav1, constitutive active Vav1 (Y174F), and the inactive (L278Q) Vav1 mutant (Razidlo *et al.*, 2014). The interaction of both Vav1 mutants to BPGAP1 was similar to that of the wild-type Vav1 (Supplemental Figure S5C). Taking the results together, the interaction of BPGAP1 with Vav1 depends on its BCH domain but is not influenced by the RacGEF activity of Vav1.

Because the formation of the BPGAP1-Vav1 complex depends on its BCH domain and the BPGAP1-Vav1 complex could be increased upon EGF stimulation, we next tested whether Vav1 binding to the BCH domain alone could be increased upon EGF stimulation. Intriguingly, the BCH-Vav1 complex had similar binding profiles in quiescent cells and in cells stimulated with EGF for 5 and 20 min (Supplemental Figure S5D), suggesting that full-length BPGAP1 could be in an autoinhibited conformation that is released upon EGF stimulation or other unknown binding partners are displaced by EGF stimulation, exposing the BCH domain for constitutively engaging Vav1.

### BPGAP1 induces cancer cell motility, invadopodium formation and cancer cell extravasation in zebrafish larvae via the RacGEF activity of Vav1

**FIGURE 5:**
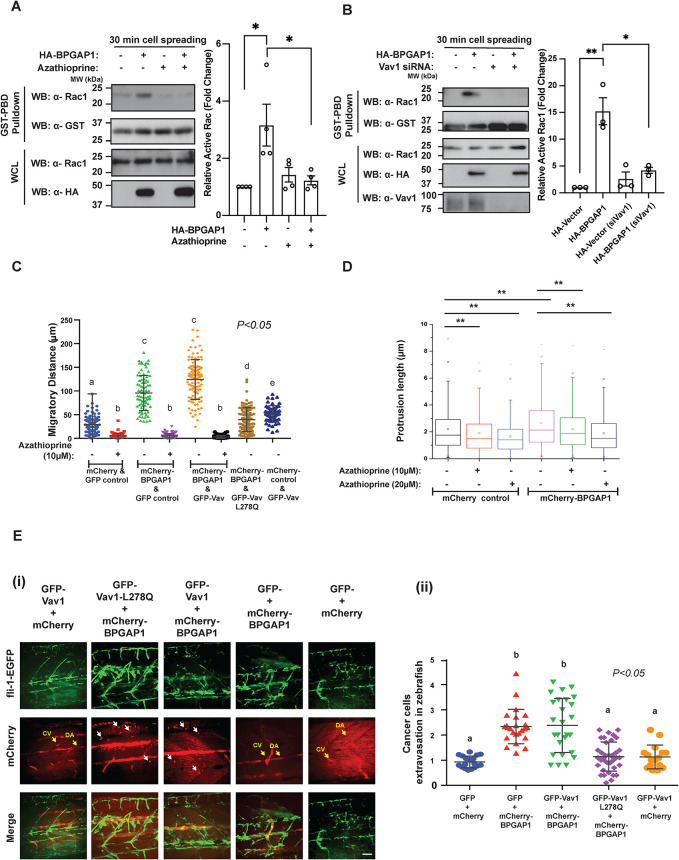
BPGAP1 induces cancer cell motility, invadopodium formation, and extravasation in zebrafish larvae via the RacGEF activity of Vav1. (A, B) Vav1 is required for BPGAP1-induced Rac1 activation*.* MCF7 cells expressing vector control or BPGAP1 were (A) treated with Vav inhibitor, azathioprine (5 μM) or (B*)* transfected with siRNA specific for Vav1. Cells were seeded on collagen-coated plates for 30 min, followed by active Rac1 pull-down assay. All data are represented as mean ± SEM (*n* = 4 [A] and 3 [B] independent experiments). ** represents *P* < 0.01, and * represents *P* < 0.05*.* A one-way ANOVA test was used to compare the different conditions to the control group. (C) Vav1 is required for BPGAP1-induced cell migration*.* MDA-MB-231 cells stably expressing wild-type and mutants of BPGAP1 and Vav1 were seeded on collagen-coated dishes and imaged by time-lapse microscopy for 2 h. Three independent experiments were performed and are represented as mean ± SEM. Data sharing different letters are statistically significant at *P* < 0.05*,* two-way ANOVA test. (D) BPGAP1 promotes longer invadopodia via Vav. MDA-MB-231-mCherry or mCherry-BPGAP1 cells were seeded on the micropit topographic features for 6 h. Cells were treated with azathioprine 2 h after seeding. All data are represented as mean ± SEM (*n* = 3 independent experiments >1000 data points). ** represents *P* < 0.01. Unpaired two-tailed *T* test. (E) BPGAP1 promotes cancer cell extravasation in zebrafish larvae via Vav1. (i) MDA-MB-231-mCherry or mCherry-BPGAP1 cells were injected into the yolk of transgenic zebrafish (fli-1-EGFP) larvae 48 hpf and fixed at 70 hpf. Cancer cell extravasation is indicated by white arrows in the representative merged images. Scale bar: 100 μm. (ii) Cancer cell extravasation was quantified. Data sharing different letters are statistically significant at *P* < 0.05, two-way ANOVA test.

To further investigate the molecular mechanism that allows BPGAP1-dependent Rac1 activation through the recruitment of Vav1, we examined the ability of BPGAP1 to induce Rac1 activation and cell motility in the presence of a Vav inhibitor, azathioprine (Razidlo *et al.*, 2014), and cells specifically depleted of Vav1. Because BPGAP1 stimulated Rac1 activity at 30 min of cell spreading (Figure 3A), this time point was used to examine whether BPGAP1-induced Rac1 activation was indeed Vav1-dependent during cell spreading. The treatment of MCF7 cells with azathioprine significantly reduced the level of active Rac1 induced by BPGAP1 (Figure 5A). Consistent with these results, the knockdown of Vav1 also reduced the BPGAP1-induced Rac1 activation (Figure 5B). Hence, BPGAP1-induced Rac1 activation during spreading depends on the activity of Vav1.

To examine whether BPGAP1-induced migration requires the RacGEF activity of Vav1, stable lines of MDA-MB-231 cells coexpressing wild-type BPGAP1 or RhoGAP mutants, together with the wild-type Vav1 or inactive Vav1-L278Q, were examined (Supplemental Figure S6A) and imaged by time-lapse microscopy. Overexpression of Vav1 alone weakly simulated cell migration, as shown by the migratory distance of the cells (Figure 5C and Supplemental Figure S6B). However, cells expressing only wild-type BPGAP1 or together with wild-type Vav1 showed enhanced cell migration (Figure 5C). In contrast, coexpression of the inactive Vav1 (L278Q) with BPGAP1 or treatment with azathioprine greatly inhibited the ability of BPGAP1 to stimulate cell migration (Figure 5C), indicating that BPGAP1-induced cell migration is dependent on active Vav1.

As BPGAP1 interacts with two signaling components that promote invadopodia, that is, Vav (Razidlo *et al.*, 2014) as reported here and cortactin (Kirkbride *et al.*, 2011), as we reported earlier (Lua and Low, 2005b), we investigated the functional role of BPGAP1 in invadopodium formation by using nano-fabricated biomaterials that mimic bone pore size. MDA-MB-231 cells expressing mCherry-BPGAP1 or control mCherry were seeded for 6 h on micropit topographic features of 2-μm width and 9-μm depth (Supplemental Figure S6, C and D). Cells extended actin-rich protrusions along the depth of the micropit features, and the length of the protrusions was quantified (Chaudhuri *et al.*, 2017). BPGAP1-overexpressing cells had significantly longer invadopodia (mean length 2.64 μm) compared with control cells (mean length 2.19 μm; *P* < 0.01) (Figure 5D). This indicates that BPGAP1 increases the invasive potential of MDA-MB-231 cells. We then examined whether the BPGAP1-induced invadopodia were dependent on Vav1 by treating the cells with the Vav inhibitor, azathioprine. Upon azathioprine treatment (10 and 20 μM), BPGAP1-overexpressing cells had a greater reduction in the length of invadopodia (Figure 5D), further supporting the notion that BPGAP1-induced invadopodium formation is dependent on Vav1 activity.

Next, we examined the effect of BPGAP1 expression on cancer cell extravasation in vivo in zebrafish, as BPGAP1 expression is associated with breast tumor metastasis to lymph nodes (Figure 1D). MDA-MB-231 cells stably expressing mCherry-BPGAP1 in the presence or absence of wild-type or mutant Vav1 were injected into transgenic fli:GFP (marker for blood vessels) zebrafish larvae (Lawson and Weinstein, 2002). Zebrafish do not develop an adaptive immune system until 14 d postfertilization. Thus, the larvae offer a rapid and robust method to evaluate the metastatic potential of human cancer cells (Nicoli and Presta, 2007; Moshal *et al.*, 2011). Consistently, higher numbers of MDA-MB-231 cells expressing BPGAP1 or coexpressing Vav1 had extravasated from the blood vessels as compared with cells expressing either vector alone, Vav1 alone, or BPGAP1 with inactive Vav1 (Figure 5E). These results further support the requirement of the RacGEF activity of Vav1 for BPGAP1-induced cell motility, invadopodium formation, and extravasation. Indeed, by screening the expression of Vav1 in tissue microarrays, we found a significant correlation of BPGAP1 and Vav1 expression in breast tumors. Similar to BPGAP1, Vav1 is detected in the cytoplasm of breast tumor epithelial cells (Supplemental Figure S6E). By Spearman analysis, the cytoplasmic level of BPGAP1 positively correlated with Vav1 in breast cancer tissues with an r-score of 0.3909 (*P* < 0.001) (Supplemental Figure S6F). We have therefore established that BPGAP1 and Vav1 cooperate to promote Rac1 activity to enhance cell motility. 

### BPGAP1 coordinates Rac1 and RhoA signaling

**FIGURE 6:**
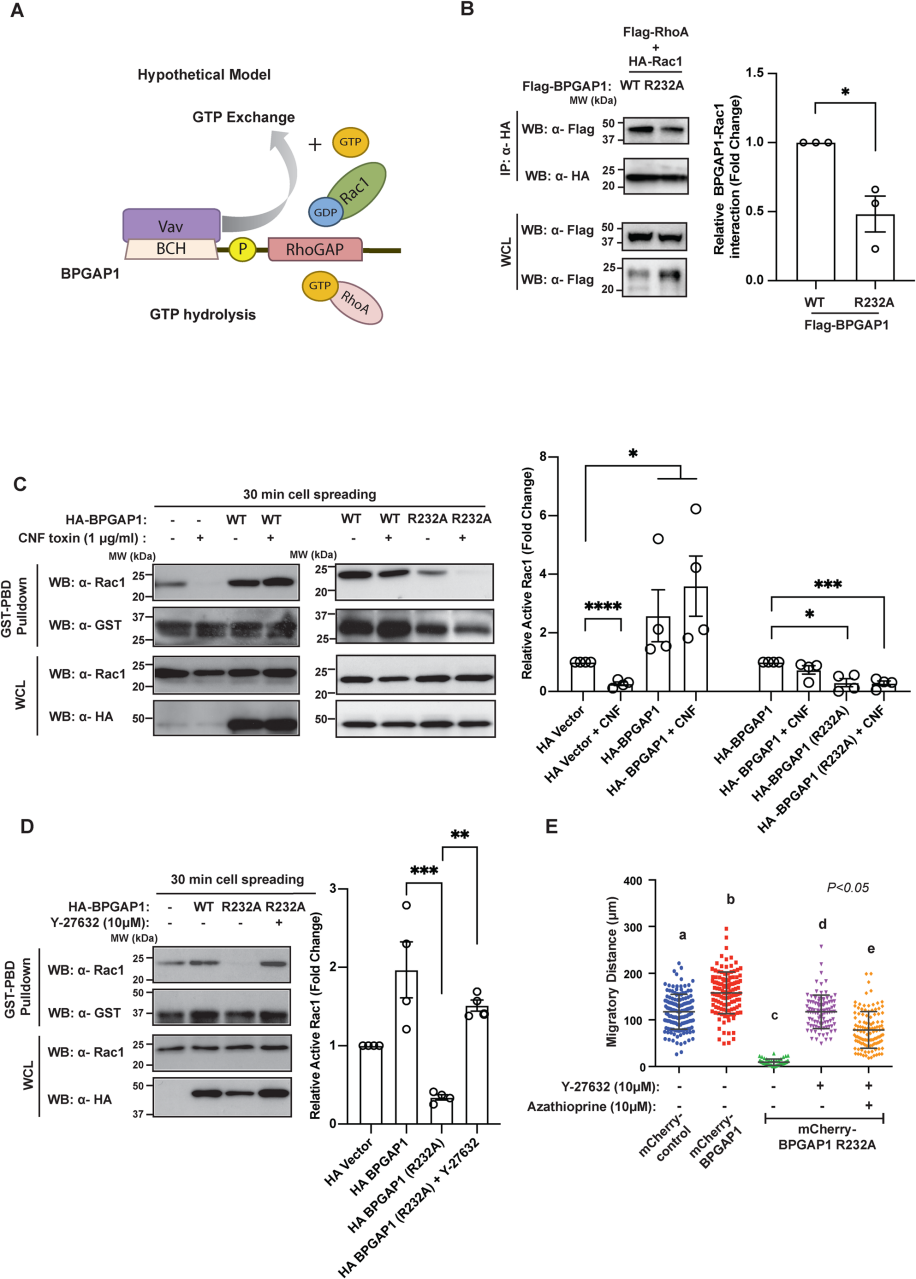
BPGAP1 coordinates Rac1 and RhoA signaling. (A) Hypothetical working model depicting BPGAP1 as a regulator for RhoA/Rac1 coupling. The possible coupling between Rac and Rho is addressed in subsequent experiments. (B) Rac1 interaction with BPGAP1 is dependent on RhoGAP activity of BPGAP1. MCF7 cells coexpressing the constructs indicated were lysed and immunoprecipitated with anti-HA beads. Bound proteins were detected by the antibodies indicated. Quantification at the side represents mean ± SEM of three independent experiments. A two-tailed Student’s *T* test was used. * represents *P* < 0.05. (C) BPGAP1-induced Rac1 activation is dependent on its RhoGAP activity. MCF7 cells expressing the indicated constructs were treated with Rho activator CNF1 toxin (CN03 1 μg/ml) for 2 h before being seeded on collagen-coated plates for 30 min. Cell were then lysed and subjected to GST-PBD beads pulldown as described earlier. Quantification at the side represents mean ± SEM of four independent experiments. * represents *P* < 0.05, *** represents *P* < 0.001, and **** represents *P* < 0.0001. (D) Downstream signaling of RhoA regulates BPGAP1-induced Rac1 activation. MCF7 cells expressing HA-vector or BPGAP1 or BPGAP1-R232A were treated with Y-27632 (10 μM) for 1 h and subjected to GST-PBD beads pull down. Quantification at the side represents mean ± SEM of four independent experiments. ** represents *P* < 0.01, and *** represents *P* < 0.001. (E) Rac and Rho coupling in BPGAP1-induced cell motility. MDA-MB-231 cells stably expressing wild-type or inactive RhoGAP mutant of BPGAP1 were treated with or without azathioprine (10 μM) and/or Y-27632 (10 μM), seeded on collagen-coated dishes, and imaged for 2 h. Please refer to Supplemental Figure S7E for time-lapse images. Three independent experiments were performed and are represented as mean ± SEM. Data sharing different letters are statistically significant at *P* < 0.05, two-way ANOVA test.

We previously showed that BPGAP1 is a RhoGAP that inactivates RhoA (Shang *et al.*, 2003). While our present findings show that BPGAP1 also serves as a scaffold that recruits Vav1 to activate Rac1, it is unclear whether both processes of RhoA inactivation and Rac1 activation occur independently via distinct mechanisms or whether they are tightly coupled to BPGAP1 (Figure 6A). To determine whether the status of RhoA activity could directly influence the ability of Rac1 to bind to BPGAP1, we examined the interaction of wild-type Rac1 with either the wild-type or the RhoGAP-inactive mutant of BPGAP1 (mutated in the catalytic arginine finger motif [Shang *et al.*, 2003]) in the presence of wild-type RhoA and active or inactive RhoA mutants. Wild-type Rac1 was used instead of Rac1-T17N, which already attained constitutively strong interaction with BPGAP1 (Figure 3B), in order to best capture possible changes in Rac1 activity (reflected in its binding) under the experimental conditions. Our results show that Rac1 interacted more strongly with wild-type BPGAP1 than with its GAP-inactive form (R232A) in the presence of wild-type RhoA in both MCF7 and HEK293T cells (Figure 6B and Supplemental Figure S7B). This result implies that either inactive GAP has directly lost its ability to recognize Rac1 or/and its defective GAP function led to the presence of active RhoA that in turn could affect Rac1 binding. Indeed, Rac1 interaction with wild-type BPGAP1 was weaker in the presence of constitutive active RhoA-G14V (which represents the active GTP-bound form of RhoA) when compared with their binding in the presence of the inactive RhoA-T19N (Supplemental Figure S7, A and B). These results support the notion that local activity of RhoA could modulate BPGAP1 interaction with Rac1. As expected, BPGAP1-R232A binding to Rac1 was also greatly affected, likely due to the indirect inhibition from the abundant level of active RhoA there. To further investigate whether inactive BPGAP1 could also affect its binding to Vav1, we examined the interaction of wild-type BPGAP1 or BPGAP1-R232A with Vav1 during cell spreading. Interestingly, both BPGAP1-R232A and wild-type BPGAP1 interacted similarly with Vav1 during cell spreading (Supplemental Figure S7C). Thus, the association of BPGAP1 and Vav1 is independent of the RhoGAP activity. Taken together, these results show that changes in the RhoGAP activity of BPGAP1 could influence the binding of Rac1 but not Vav1, likely due to the local activity status of RhoA, which we went on to investigate.

Because the RhoGAP activity of BPGAP1 on RhoA influences the binding and activation of Rac1, we sought to determine how activating or inactivating RhoA signaling could directly affect BPGAP1-induced Rac1 activation. To do this, we used CNF1 toxin (Knust and Schmidt, 2010) as a stimulator of Rho and compound Y-27632, which inhibits ROCK (Rho-associated protein kinase), an effector of active Rho. Both the control and BPGAP1-expressing cells were treated with or without CNF1 toxin, and Rac1 activity was determined after 30 min of cell spreading. The efficacy of CNF1 toxin in activating RhoA was first verified (Supplemental Figure S7D). As expected, Rac1 activity was reduced in control cells treated with CNF1 toxin that had activated RhoA. However, wild-type BPGAP1 through its ability to inactivate RhoA prevented the reduction of Rac1 activity that was induced by CNF1 toxin (Figure 6C, left panel). In contrast, BPGAP1-R232A, which could not inactivate RhoA, failed to antagonize the effect of CNF toxin in the activation of RhoA. Thus, Rac1 remained inactive (Figure 6C, right panel). This result further supports the requirement of BPGAP1 to inactivate RhoA for Rac1 activation by allowing Rac1 binding to BPGAP1.

Next, we examined whether inhibiting downstream RhoA signaling can rescue the effect of BPGAP1-R232A that reduced Rac1 activity through active RhoA. Indeed, treatment of cells with the ROCK inhibitor Y-27632 effectively restored Rac1 activation at 30 min of cell spreading (Figure 6D). This result indicates that RhoA/ROCK signaling, when active, provides negative feedback to reduce BPGAP1-induced Rac1 activation. Taken together, these results further support a concerted mechanism in which BPGAP1-induced Rac1 activation is dependent on the RhoGAP activity of BPGAP1 and that BPGAP1 itself could directly regulate the dynamic and reciprocal nature of the antagonistic Rho/Rac signaling.

We further investigated the Rac1/RhoA coupling by BPGAP1 in cell motility by using the inactive BPGAP1 mutant. A stable MDA-MB-231 cell line expressing wild-type mCherry-BPGAP1 or mCherry-BPGAP1-R232A was generated in the absence of BPGAP1 (Supplemental Figure S2C). While overexpression of mCherry-BPGAP1 significantly promoted polarization and migration of MDA-MB-231 cells, BPGAP1-R232A strongly reduced MDA-MB-231 cell migration (Figure 6E; Supplemental Figure S7E). To test the corequirement of Rho and Rac regulation by BPGAP1 for cell motility and to overcome the migratory defect of BPGAP1-R232A–expressing cells, we treated these cells with the ROCK inhibitor Y-27632, which completely restored cell migration (Figure 6E and Supplemental Figure S7Eii). These results indicate that the RhoGAP activity of BPGAP1 is important for promoting cell migration. In contrast, cotreatment of BPGAP1-R232A cells with both Y-27632 and the Vav inhibitor, azathioprine, essentially blocked the rescue of migration by Y-27632 (Figure 6E and Supplemental Figure S7Eii). Therefore, tight coordination of RhoA and Rac1 activities by BPGAP1 and Vav is crucial in promoting cell motility.

**FIGURE 7:**
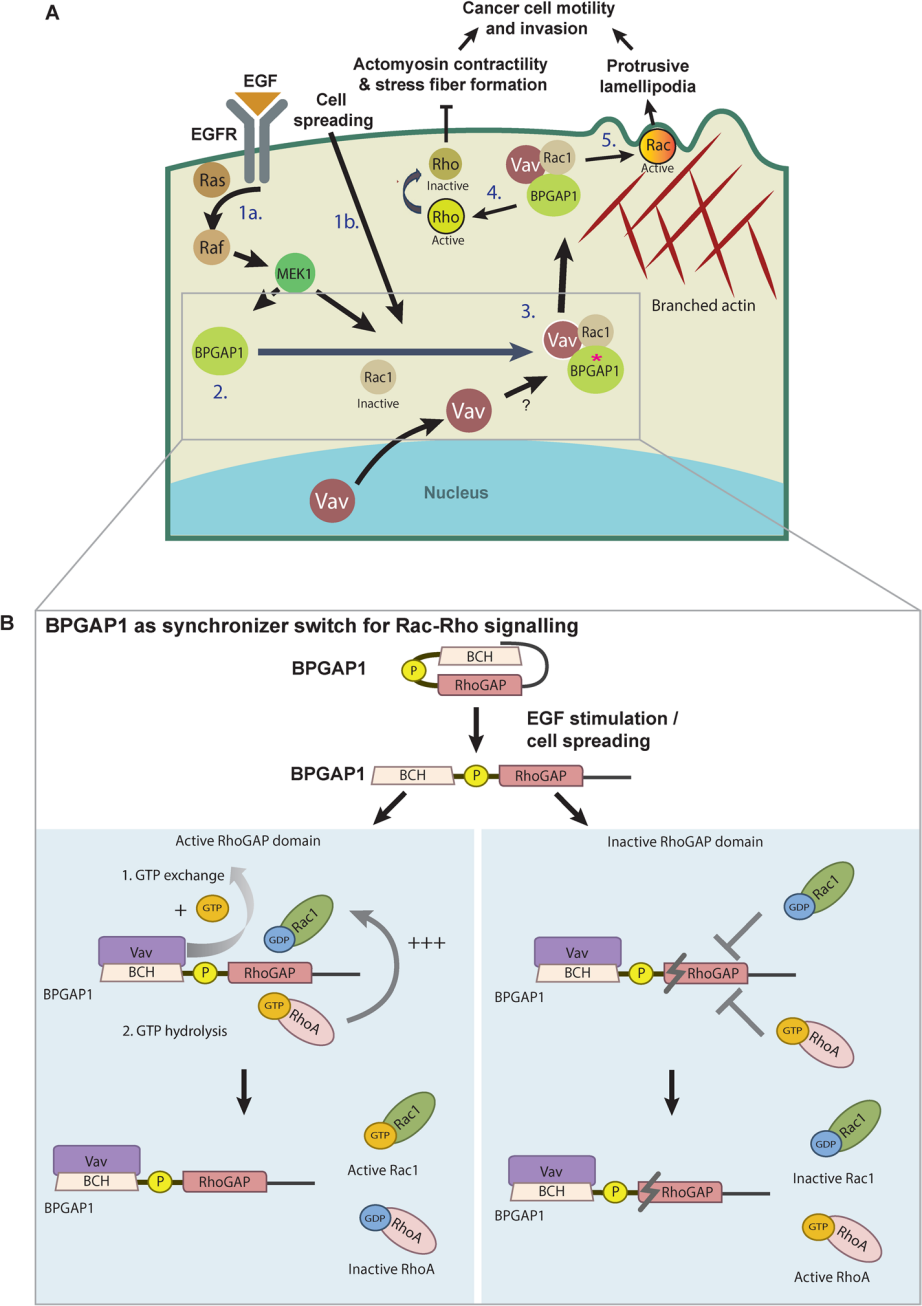
Schematic model depicting BPGAP1 as a switch that synchronizes Rac and Rho signaling during cancer cell motility and invasion. (A) Model for how BPGAP1 synchronizes Rho/Rac signaling during cell motility. The autoinhibited BPGAP1 is released upon growth factor stimulation and cell spreading (step 1). BPGAP1 recruits Rac1 and Vav1 (step 2; asterisk represents the “open conformation” of BPGAP1) to activate Rac1 (step 3) while inactivating RhoA, leading to reduced actomyosin contractility (step 4) and enhanced protrusive lamellipodia that collectively drive cancer cell motility and invasion (step 5). (B) BPGAP1 synchronizes Rac1/RhoA signaling. Upon growth factor stimulation and cell spreading, active BPGAP1 promotes Rac1 activation and RhoA inactivation (left panel), whereas the inactive BPGAP1 maintains a high level of RhoA activity that also impairs Rac1 binding to BPGAP1, thus preventing Rac1 from being activated (right panel). Please refer to the text for more details. “T” symbol refers to inhibition and the symbol refers to inactive RhoGAP.

In summary, our results highlight the importance of BPGAP1 as a unique *placemaker* that recruits Vav1 and Rac1 to promote Rac1 activation for cell motility. It also serves as a crucial *pacemaker* that controls the timing of Rac1 activation with RhoA inactivation via its RhoGAP activity. Our findings reveal an unprecedented mechanism by which a single RhoGAP, BPGAP1, not only functions to inactivate RhoA but also acts as a dual-function scaffold protein that locally recruits Vav1 to activate Rac1 in a highly synchronized spatiotemporal manner to drive cell migration (Figure 7A). This molecular switch synchronizes the activities of Vav1, RhoA, and Rac1 through its distinct BCH and RhoGAP domains. In addition, the coupling of Rac1 and RhoA activities is further regulated by its RhoGAP activity (Figure 7B). Consequently, loss of either Rac1 or RhoA regulation by BPGAP1 impairs cell spreading and cell migration.

## DISCUSSION

Cell signaling involves a highly complex and well-regulated network of proteins that specifically detect and transmit signals (messengers/molecular switches) and execute the effects and outcomes (effectors). Central to this intricate control are groups of regulators that modify the activities of these switches. For Rho small GTPases such as Rho and Rac, their activator GEFs and inactivator GAPs are critical in controlling their cellular activities and, therefore, outcomes. However, little is known about where, when, and how these crucial regulators themselves are coordinated in concert.

Here, we have revealed a novel mechanism whereby RhoA inactivation by its RhoGAP BPGAP1 directly influences Rac1 binding to BPGAP1 for its subsequent activation by the BPGAP1/Vav complex. Therefore, BPGAP1 not only serves as a functional scaffold that recruits Vav to activate Rac1, but it also serves as a common checkpoint that coordinates and integrates the activation and inactivation cycles of Rac1 by directly modulating the local activity of RhoA. Interestingly, the RhoGAP-inactive mutant of BPGAP1 could interact with Vav1, but it fails to promote Rac1 activation, suggesting that BPGAP1 binding to Vav1 alone is insufficient to activate Rac1. Instead, the binding of BPGAP1 to Rac1 is dependent on the status of RhoA activity. Another possibility is that the local level of RhoA activity could separately regulate the RacGEF activity of Vav, thus indirectly impacting Rac1 activation. The detailed structural mechanism(s) by which the activity and binding of RhoA influence Rac1 binding to BPGAP1 and how inactive BPGAP1 intrinsically reduces Rac1 binding require further investigation.

By coordinating Rac1 and RhoA coupling, BPGAP1 serves as a regulatory switch to synchronize the timing of RhoA inactivation with Rac1 activation. Both the recruitment of Vav and the activity of its RhoGAP domain are regulated by the release of an autoinhibitory switch in a process that is likely to involve multiple mechanisms (e.g., phosphorylation) upon EGF stimulation and cell adhesion. Two kinases downstream of EGF receptor signaling have been implicated to have such potential effects. First, MEK2 is reported to relieve the autoinhibited BPGAP1 via its action on the proline-rich motif proximal to the RhoGAP domain (Pan *et al.*, 2010). Second, the phosphorylation of BPGAP1 by JNK could also release the autoinhibition to allow BPGAP1 to promote MEK/MP1 activation (Jiang *et al.*, 2016). We recently also showed that in p50RhoGAP/Cdc42GAP (a homologue of BPGAP1), its RhoGAP activity toward RhoA is activated upon release of its autoinhibition between its BCH domain and GAP domain (Chichili *et al.*, 2021). All these findings indicate signaling cross-talk involving BPGAP1, not just between Rho/Rac but also between Rho GTPases and MAPK kinase. By contrast, BPGAP1 displayed similar binding profiles with wild-type Vav1 and the Vav1 mutants, suggesting that the interaction of BPGAP1 with Vav is likely to be constitutive once their interaction is induced upon EGF stimulation. This would ensure proper timing of Rac activation.

Interestingly, BPGAP1 can regulate the cellular localization of Vav1 by inducing Vav1 translocation from the nucleus to the cytoplasm in the absence of BPGAP1 nuclear shuttling. Vav1 is known to interact with transcription factors and regulates gene expression (Movilla and Bustelo, 1999; Houlard *et al.*, 2002; Lee *et al.*, 2008), and its DH-PH domain contains a nuclear localization signal (NLS), suggesting a possible role of Vav1 as a transcription coregulator (Kranewitter and Gimona, 1999). It remains to be seen whether BPGAP1 might prevent Vav1 from entering the nucleus by masking its NLS and instead shuttling it to lamellipodia to regulate cell motility. Importantly, our results also point to the versatile role of BCH domains in scaffolding GEFs and GTPases. We recently showed that BNIP-2, through its BCH domain, engages RhoGEFH1 to activate RhoA upon microtubule depolymerization (Pan *et al.*, 2020) and activates Rho-dependent cellular contractility that leads to BNIP-2 scaffolding YAP for its inactivation by LATS (Wong *et al.*, 2022). Therefore, it is tempting to speculate that the BCH domain of BPGAP1 could act similarly, recruiting a RhoGEF to reactivate RhoA (analogous to its ability to activate Rac via Vav1) after the initial cycle of RhoA inactivation by the adjacent RhoGAP domain. Synchronizing Rho activation/inactivation cycles with Rac activity locally would ensure rapid responses to external and internal signals. Earlier work has shown that p190GAP can act as a RacGAP or a RhoGAP, depending on lipid binding and/or phosphorylation (Ligeti *et al.*, 2004; Levay *et al.*, 2009, 2013), thus offering important contextual control on the spatiotemporal activity of Rho and Rac. It would be interesting if BPGAP1 could also directly or indirectly undergo contextual fine-tuning to inactivate Rac in addition to Rho.

Besides its elevated expression across all stages of breast cancer and its association with HER+ breast cancer, significantly higher expression of BPGAP1 is also detected in lung, pancreas, cervix, colon, ovary, and stomach cancers, implying that it plays a role in different types and stages of tumor progression. The current data that show BPGAP1’s effect in promoting phenotypes associated with metastasis are all consistent with its contributions to epidermal growth factor receptor (EGFR)/Ras/MAPK signaling (Lua and Low, 2005a; Pan *et al.*, 2010; Jiang *et al.*, 2016) and coordinating Rho/Rac signaling that we reported here. Therefore, the present findings highlight the versatility of this RhoGAP in controlling cancer cell motility, consistent with a role in cancer progression. As EGF receptor, Vav1, RhoA, Rac1, and BPGAP1 are all associated with oncogenesis and/or cancer metastasis, the identification of this novel EGFR-BPGAP1-Vav1-Rac1-RhoA signaling axis could provide new approaches to a combinatorial therapeutic intervention in cancer progression.

## MATERIALS AND METHODS

Request a protocol through *Bio-protocol*.

### Cell culture and transfection

All cell lines used in this study were purchased directly from the American Type Culture Collectionand routinely checked (quarterly) for mycoplasma contamination with the MycoStrip–Mycoplasma Detection Kit (InvivoGen) or the MycoAlert PLUS Mycoplasma Detection Kit (Lonza). HEK293T cells were maintained in RPMI-1640 (Hyclone) with 10% fetal bovine serum (FBS), 2 mM l-glutamine, 100 U/ml penicillin, and 100 μg/ml streptomycin (Hyclone). MCF7 and MDA-MB-231 cells were grown in high-glucose DMEM (Hyclone), 10% FBS, 100 U/ml penicillin, and 100 μg/ml streptomycin (Hyclone). All cells were grown at 37°C, 5% CO_2_. HEK293T and MCF7 cells were transfected using Mirus (TransIT), while MDA-MB-231 cells were transfected using Lipofectamine 2000 (Invitrogen). EGF stimulation (100 ng/ml EGF; Sigma) was performed on quiescent cells (starved for 18 h in serum-free medium).

### Plasmid construction and mutagenesis

All human BPGAP1, cortactin, Rac1, and RhoA (gift from Late Alan Hall, Memorial Sloan-Kettering Cancer Center) constructs were cloned in Flag-, HA-, GFP-, and mCherry PXJ40 vectors (gift from Ed Manser, IMCB, Singapore). The HA-tagged human wild-type and mutants of Vav1 were a generous gift from Mark A. McNiven (Mayo Clinic). The wild-type and mutants of Vav1 were subcloned into GFP-tagged PXJ40 vectors. GST-Rhotekin-RBD was a generous gift from Simone Schoenwaelder (Monash University, Australia), and GST-PBD was a gift from Trina Schroer (Johns Hopkins University, Baltimore, MD; Addgene plasmid #60880).

### Coimmunoprecipitation and Western blot analyses

Cells were lysed with RIPA buffer (150 mM NaCl, 50 mM Tris-HCl, pH 7.3, 0.25 mM EDTA, 1% [wt/vol] sodium deoxycholate, 1% [vol/vol] Triton X-100, 50 mM NaF, 5 mM sodium orthovanadate, protease inhibitors [Roche Applied Science]). Cell lysates with overexpressing Flag-tagged or HA-tagged proteins were immunoprecipitated with anti-HA magnetic beads (Thermo Scientific) or anti-Flag M2 affinity gel beads (Sigma), as previously described (Low *et al.*, 2000). For immunoprecipitation of endogenous BPGAP1, lysate was incubated with immunoglobulin (Ig) control or anti-BPGAP1, followed by protein A/G Sepharose beads (GE Healthcare). Bound proteins and lysate proteins were separated in SDS–PAGE gels, transferred to polyvinylidene difluoride (PVDF) membranes and probed with polyclonal anti-HA (Zymed), polyclonal anti-Flag (Sigma), polyclonal anti-RhoA (Santa Cruz), monoclonal Rac1 (BD Transduction Laboratories), monoclonal phospho-ERK (Sigma), monoclonal pan-ERK1/2 (BD Transduction Laboratories), monoclonal dsRed, polyclonal GFP (Santa Cruz), monoclonal GAPDH (Santa Cruz), polyclonal GST (in-house produced), polyclonal Vav (Santa Cruz), or polyclonal BPGAP1 (in-house produced) antibodies. For quantification of Western blots, the densitometric values of pull-down bands were divided by the total pull-down band densitometric value. This value was then normalized to the total protein densitometric value quantified from the whole cell lysate (WCL). All Western blots were repeated at least three times.

### Generation of stable cell lines

MDA-MB-231 cells were transfected with constructs encoding various fluorescent protein–tagged proteins. The cells were selected using cell sorting with a FACS-AriaII. Single cells were plated into wells in a 96-well plate, and single stable colonies expressing the fluorescent protein–tagged proteins were selected. These stable clones were expanded and protein expression verified by immunoblotting.

### RNA interference

The BPGAP1 RNA interference (RNAi) target sequence 5′-CGCATACAAGGAGTTCGAT-3′ was designed (using Invitrogen Block-iT RNAi designer) and cloned into pGFP-V-RS vector (Origene) as a short-hairpin RNAi BPGAP1 construct. The short interfering RNA (siRNA) target sequences for BPGAP1 (5′-GCACGAGUCACCCGUUCUA-3′) and Vav1 (5′-GGACCUGCUUCGUGUUCAU-3′) were designed by Invitrogen Block-iT RNAi designer and synthesized by Sigma.

### Cell spreading assay

Cell culture dishes (35 mm) or six-well plates were coated with 10 μg/cm^2^ rat tail collagen Type I (Sigma) for 1 h at 37°C. The collagen-coated dishes or wells were washed with phosphate-buffered saline (PBS) and dried. Cells (5 × 10^4^) were seeded on the collagen-coated dishes or wells. The cells were imaged using a Nikon Biostation IMQ or harvested at the indicated times.

### Immunofluorescence

Cells on 25-mm glass coverslips were fixed with 4% paraformaldehyde in PBS at room temperature for 15 min. The coverslips were washed three times with PBS and permeabilized with 0.2% Triton X-100 in PBS at room temperature for 15 min, followed by blocking with 2% bovine serum albumin and 7% FBS in PBS for 1 h. The cells were then incubated with 40 μl of blocking buffer containing 0.4 μg of primary antibody for 1 h at room temperature. The primary antibodies were polyclonal anti-Flag (Sigma), anti-BPGAP1 (in-house produced), and anti-active Rac1 (NewEast Biosciences). Samples were washed three times with PBS containing 0.1% Triton X-100 and incubated with various Alex Fluor–conjugated secondary antibodies (Invitrogen) for 1 h at room temperature. The samples were further washed three times with PBS containing 0.1% Triton X-100 before being mounted with FluorSave (Calbiochem) for image acquisition.

### Reverse transcription-PCR

For RT-PCR, mRNA was purified using an RNeasy Mini Kit (Qiagen) and cDNA was synthesized using SuperScript III Reverse Transcriptase (Invitrogen). BPGAP1 was amplified using a DyNAzyme PCR Kit (Thermo Fisher Scientific) with primer pairs: 5′-tacctgagtgagctccacgaa-3′ and 5′-aggaaggtcttcaggatcacg-3′. GAPDH was amplified with primer pairs: 5′-gagtcaacggatttggtcgt-3′ and 5′-ttgattttggaggg­atctcg-3′. Amplification of cDNAs was performed using PCR with the thermal cycling conditions as follows: activation at 95°C for 2 min, followed by 30 cycles of denaturing at 95°C for 30 s, annealing at 54°C for 30 s, and extension at 72°C for 30 s with the final extension at 72°C for 10 min. Vav1 was amplified with primer pairs: 5′-tgaaacacacgcaggaggcga-3′ and 5′-ccatagtgagccagagactgg-3′. Vav2 was amplified with primer pairs: 5′-tgctcaagtcccacgccagc-3′ and 5′-ccagctgcttgaagctctcc-3′. Vav3 was amplified with primer pairs: 5′-gcacaggaccaaagagtcag-3′ and 5′-cccctctgtccagctgaatg-3′. Amplification of cDNAs was performed using PCR with thermal cycling conditions as follows: activation at 95°C for 30 s, followed by 50 cycles of denaturing at 95°C for 5 s and annealing at 62°C for 10 s.

### Drug treatment

Cells were treated where indicated with 10 μM U0126 (Cell Signaling Technology), 10 μM Y-27632 (Sigma), and 10 μM azathioprine (Sigma) for 1 h at 37°C; 1 μg/ml CNF toxin (#CN03; Cytoskeleton) was added to cells for 2 h at 37°C.

### Rac1 and RhoA activation assay

Cells were lysed in RIPA buffer, and 300 μg lysates were incubated with 20 μg of recombinant GST-Rhotekin-RBD (generous gift of plasmid from Simone Schoenwaelder, Monash University, Australia) or GST-PBD (generous gift of plasmid from Trina Schroer [Johns Hopkins University, Baltimore, MD; Addgene plasmid #60880]) on glutathione beads for 30 min at 4°C. The beads were washed three times with RIPA buffer. Bound and lysate proteins were analyzed by Western blotting with anti-RhoA (Santa Cruz) and anti-Rac1 (BD Transduction Laboratories) antibodies.

### Invadopodium formation assay

Micropit topographic features were produced using SU-8 on silicon wafers. Polydimethylsiloxane (PDMS; Sylgard 184; Dow Corning) was mixed uniformly with curing agent in a 10:1 ratio and poured on the silicon wafer. It was then allowed to solidify at 80°C for 2 h and solidified PDMS was peeled off from the silicon wafer. The PDMS topographic features were made hydrophilic by treatment with air-plasma for 3 min (Model PDC-002; Harrick Scientific Corp.) and UV sterilized for 5 min in the presence of 70% isopropanol. The PDMS features were washed three times with sterile PBS and coated with 10 μg/cm^2^ rat tail collagen Type I (Sigma) for 1 h at 37°C. Subsequently, the PDMS features were washed with PBS three times and 70,000 cells were seeded on each feature. Cells were cultured at subconfluence for 6 h. For the drug treatment assay, the cells were seeded for 2 h before treatment with 10 or 20 μM azathioprine (Sigma) for 4 h. Subsequently, cells were fixed with 4% paraformaldehyde and permeabilized with 0.01% Triton X-100. F-actin was then stained using Alexa Fluor 488 phalloidin (Invitrogen) and imaged using a Nikon Confocal A1R microscope. Protrusion length was quantified by selecting the region of interest along the *z*-axis using the image analysis software Imaris (Bitplane, Zurich, Switzerland). Approximately 300 cells were quantified per experiment. 

### Cancer metastasis assay in zebrafish larvae model

Transgenic zebrafish (fli-1-EGFP) embryos were incubated at 28°C in egg water. At 48 h postfertilization (hpf), zebrafish embryos were anesthetized with 0.016% tricaine and transferred onto agar plates for microinjection. Fifty MDA-MB-231 cells suspended in 9.6 nl of serum-free DMEM were injected into the yolk of each embryo using a microinjector (World Precision Instruments, UK). The injected zebrafish embryos were individually transferred to single wells of 12-well plates with 1 ml of egg water. The zebrafish embryos were incubated at 28°C for 70 h. The embryos were fixed in 4% paraformaldehyde overnight at 4°C and imaged using a Perkin Elmer confocal spinning disk. All experiments and maintenance of zebrafish were in compliance with the approved protocols by the NUS Institutional Animal Care and Use Committee.

### Image analysis

The analysis of nucleus-to-cytoplasmic ratio (N/C ratio) of Vav was processed by an in-house developed semiautomated algorithm written in MATLAB (Mathworks, USA). Both nuclear and cytoplasmic regions were manually selected according to the signal distribution on the Z-maximum projected images. Maximum projected masks for nuclei and cytoplasmic regions were then generated. These were used to multiply the entire Z-stack of the nuclear and cytoplasmic regions to remove the background noise that would possibly undermine the segmentation accuracy. The mean and SD of intensity was computed throughout the entire Z-stack for 3D segmentation. The criteria for setting a pixel as 1 or 0 was based on the mean ± (SD × *a*), where *a* is a value to adjust the criteria. Pixels above mean ± (SD × *a*) were set as 1, and those below were set as 0. The segmentation procedure was monitored by merging the outline of the segmented object with the original object. This ensures optimal 3D masking for both nuclear and cytoplasmic regions, which is critical for the quantification. The N/C ratio of Vav1 was then calculated by dividing the total nuclear level by the cytoplasmic level of Vav1 after background subtraction.

### Tissue scan cDNA array

Breast cancer cDNA array II (Gene Technologies, Rockville, MD) was used to perform real-time RT-PCR to investigate the mRNA expression of BPGAP1 in breast cancer tissue samples. The array consisted of cDNAs from 48 breast tissues with different stages, including 5 normal, 11 Stage I, 8 Stage IIA, 6 Stage IIB, 8 Stage IIIA, 2 Stage IIIB, 4 Stage IIIC, and 4 Stage IV tumors. Amplification of the cDNAs was performed using SYBR Green-based, real-time RT-PCR according to the manufacturer’s protocol. The stock solution contained 2× QuantiTect SYBR Green PCR master mix, forward and reverse primers for BPGAP1, and RNase-free water. Real time RT-PCR was performed using Applied Biosystems’ 7500 Real-Time PCR Systems, and the thermal cycling conditions were as follows: activation at 95°C for 15 min, followed by 40 cycles of denaturing at 94°C for 15 s, annealing at 60°C for 30 s, and extension at 72°C for 60 s.

### Statistical analysis from TCGA data

Statistical analysis of BPGAP1 expression in breast cancer patients was performed using GENT and UALCAN portals. For UALCAN portal data, the raw values were downloaded from the UALCAN portal and then plotted with GraphPad Prism. One-way analysis of variance (ANOVA) was used to compare different conditions to the control; Gaussian distribution was assumed, and the Greisser–Greenhouse correction was used to account for sphericity differences. For BPGAP1 and ER status immunostaining analysis, data from the Cancer Genome Atlas (TCGA) were downloaded using cBioPortal. Outliers were removed using the ROUT (Q value = 1.000%) function of GraphPad Prism followed by calculations of *P* values using *T* tests. A *P* value of <0.05 was considered to be statistically significant. For the BPGAP1 expression and BRCA-specific receptors with distant metastasis-free patient survival data from TCGA, Kaplan–Meier survival plots were plotted using the KM plotter server, and a logrank test was performed to compare survival curves.

### Patient cohorts of the breast tissue microarrays

Pathologically diagnosed breast cancer tissue samples (197) were obtained from the Department of Pathology, Singapore General Hospital, between 1998 and 2004. The study protocol was reviewed and approved by the Institutional Review Board. Tissue microarrays (TMAs) were constructed using a Beecher arrayer. Clinicopathological features of the patients including age, race, tumor size, lymph node status, estrogen, progesterone and HER2 receptor status, histological grade, and the extent and grade of ductal carcinoma in situ (DCIS) were collected for association study. The age of patients ranged from 33 to 85 yr, with a mean age of 54.1 yr. The patient cohorts consisted predominantly of Chinese, which made up 80.7% of total cases, with the remaining cases including Malays (8.1%), Indians (4.1%), and other ethnic groups (7.1%). The demographic distribution of clinical parameters is shown in Table 1. For the analysis of BPGAP1 expression, 30 sections were excluded during processing, with the remaining 167 cases proceeding to examination.

**TABLE 1:** Frequency distribution of breast cancer patients used for BPGAP1 and VAV1 study.

Clinicopathological parameters	Frequency distribution, *n* (%)
Age (yr)	
Mean	54.1
Minimum	33
Maximum	85
Race	
Chinese	159 (80.7)
Malay	16 (8.1)
Indian	8 (4.1)
Others	14 (7.1)
Tumor size (in mm)	
≤20	50 (25.4)
>20	144 (73.1)
Not available	3 (1.5)
Histological grade	
G1	18 (9.1)
G2	73 (37.1)
G3	93 (47.2)
Not available	13 (6.6)
Lymph node stage	
0	90 (45.7)
1	53 (26.9)
2	31 (15.7)
3	16 (8.1)
Not available	7 (3.6)
Tubule formation	
1	11 (5.6)
2	61 (31.0)
3	111 (56.3)
Not available	14 (7.1)
Pleomorphism	
1	5 (2.5)
2	83 (42.1)
3	95 (48.2)
Not available	14 (7.1)
Mitotic index	
1	40 (20.3)
2	59 (29.9)
3	84 (42.6)
Not available	14 (7.1)
DCIS extent	
None	47 (23.9)
Minimal	72 (36.5)
Intermediate	1 (0.5)
Extensive	22 (11.2)
Not available	55 (27.9)
DCIS grade	
Low	8 (4.1)
Intermediate	45 (22.8)
High	63 (32.0)
Not available	81 (41.1)
ER	
Negative	67 (34.0)
Positive	129 (65.5)
Not available	1 (0.5)
PR	
Negative	97 (49.2)
Positive	99 (50.3)
Not available	1 (0.5)
HER2	
Negative	115 (58.4)
Positive	74 (37.6)
Not available	8 (4.1)
ER/PR/HER2	
Negative	30 (15.2)
Positive	159 (80.7)
Not available	8 (4.1)

ER, estrogen receptors; PR, progesterone receptors.

### Immunohistochemical staining of breast tissue microarrays

Immunohistochemical staining of TMAs was performed with BPGAP1 (in-house production) and VAV1 antibodies (Acris-OriGene; Catalogue: AP06359PU-N) using the Bond Max automated immunohistochemistry vision Biosystem (Leica Microsystems GmbH, Germany) based on the manufacturer’s protocol. TMAs were first deparaffinized, and antigen retrieval was conducted by boiling the slides with Bond Epitope Retrieval Solution 2 (citrate-based pH 9.0 solution) at 98°C for 20 min. This was followed by peroxidase blocking using the Bond Polymer Refine Detection Kit DC9800 (Leica Microsystems GmbH, Germany). TMAs were washed once before incubation with the BPGAP1 (1:50) or VAV1 (1:100) antibodies for 30 min. Finally, polymer was added, and the staining color was developed using DAB-Chromogen.

### Immunohistochemical assessment of breast TMAs

The tissue slides were scanned using the ScanScope System (Aperio Technologies, Vista, CA), and ImageScope software was used to view the slides. BPGAP1 and VAV1 staining was scored using a semiquantitative method. Immunoreactivity was assessed in the cytoplasm of breast cancer epithelial cells, and the staining intensity was denoted as 0 (absent), 1+ (weak), 2+ (moderate), and 3+ (intense). The percentage of cells stained was recorded, and the IRS was calculated as ∑(intensity*_n_* × percentage of positive stained cells with intensity*_n_*). The staining results were validated by a researcher and confirmed independently by a pathologist with both blind to the clinical data.

### Statistical analysis

Statistical analysis for immunohistochemical staining was performed using PASW Statistics 18 software (SPSS) for Windows and STATA version 10 software (STATACorp LP, USA). The staining difference for BPGAP1 or VAV1 between the adjacent normal and tumor tissues was analyzed using a nonparametric Mann–Whitney test. Univariate analysis was performed using either a Fisher exact test or a Chi-square test as appropriate to investigate the association between BPGAP1 or VAV1 expression with clinicopathological features, respectively. For BPGAP1 and VAV1 immunostaining correlation analysis, Spearman’s nonparametric correlation was used. For tissue scan cDNA array results, GraphPad Prism software version 5.0 (San Diego, CA) was used for statistical analysis. For statistical differences comparing immunoblots, the data are assumed to be approximately normal, and one-way ANOVA was used to compare more than two conditions to the control and an unpaired Student’s *T* test was used to compare two groups. Unless otherwise stated, data are presented as mean ± SEM. Wherever appropriate, the number of replicates and statistical values are indicated in each figure legend. * represents *P* < 0.05, ** represents *P* < 0.01, *** represents *P* < 0.001, and **** represents *P* < 0.0001.

## Abbreviations used:

BCH: BNIP-2 and Cdc42GAP homology
BPGAP1: BNIP-2 and Cdc42GAP Homology (BCH) domaincontaining, Proline-rich and Cdc42GAP-like protein subtype-1
EGFR: epidermal growth factor receptor
ER: estrogen receptors
GAP: GTPase-activating protein
GEF: guanine nucleotide exchange factor
GENT: gene expression database of normal and tumor tissues
HER2: human epidermal growth factor receptor 2
PR: progesterone receptors
UALCAN: University of Alabama at Birmingham Cancer data analysis Portal.
