# Supplementary material for: The scaffold RhoGAP protein ARHGAP8/BPGAP1 synchronizes Rac and Rho signaling to facilitate cell migration
Source: Mol Biol Cell. 2023 Feb 21;34(3):ar13. doi: 10.1091/mbc.E21-03-0099 (PMC10011724; doi:10.1091/mbc.E21-03-0099)

Supplementary Materials

Molecular Biology of the Cell

Wong *et al*.

Supplementary Figure Legends

Figure S1. BPGAP1 expression is elevated in breast cancer. *BPGAP1 expression is highly upregulated in breast cancer.* Through online GENT analysis (http://medicalgenome.kribb.re.kr/GENT/), BPGAP1 expression is most abundant in breast cancer samples and its expression is elevated in breast tumour (breast-C) (red arrow) compared to the normal tissues (breast-N).

Figure S2. Stable BPGAP1-expressing breast cancer cell line is generated to study its pro-metastatic role. ***(A)*** *Genotyping mRNA expression of BPGAP1 in breast cancer cell lines (MCF7 and MDA-MB-231) by* RT-PCR analysis, n=4 ***(B)*** *BPGAP1 expression profile in breast cancer cell lines.* Western blot analysis of BPGAP1 expression in MCF7 and MDA-MB-231 cells. Loading control represents MDA-MB-231 cells transfected with Flag-BPGAP1. n =3. ***(C)*** *Stable expression of vector control and BPGAP1.* Western blot analysis of MDA-MB-231 cells stably expressing either vector control or BPGAP1.

Figure S3. BPGAP1 promotes the migration of MDA-MB-231 breast cancer cells. ***(A)*** *BPGAP1 promotes cell polarization and motility.* MDA-MB-231 cells stably expressing mCherry vector control or mCherry-BPGAP1 were seeded on collagen-coated dishes. Time-lapse images of cells were acquired for 45 minutes. Representative images are shown; Scale bar: 70 μm. Red square highlights lamellipodia structures at protrusion ends. ***(B)*** Cell aspect

ratio, ***(C)*** migration tracks (2 representative tracks are illustrated as black and red that were tracked over 2 hours), ***(D)*** total distance migrated (over 2 hours) and ***(E)*** speed of migration (tracked over 2 hours) were quantified using ImageJ and plotted using Matlab. All the data above were obtained from 3 independent experiments and represented as mean ± SEM. *N.S.*, not significant; *** represents *P<0.001*; **** represents *P<0.0001*, One Way ANOVA test.

Figure S4. BPGAP1 interacts with Vav and re-localises Vav from nucleus to cytosol and lamellipodia. ***(A)*** *BPGAP1 interacts with Vav1, Vav2 and Vav3.* HEK293T cells were co-transfected with Flag-BPGAP1 and HA-Vav1, Vav2 or Vav3. Cells were lysed and immunoprecipitated with anti-Flag beads. Both bound and WCL proteins were analysed by immunoblotting, n=3. ***(B)*** Online cell line database shows expression all Vav isoforms in MCF7 cells, (Human Protein Atlas proteinatlas.org). The graph represents box plot showing minimum to maximum values of Vav isoform FPKM. ***(C)*** *Genotyping of Vav isoform mRNA expressions of in MCF7 and MDA-MB-231 breast cancer cell lines*, n=3. ***(D)*** *BPGAP1 re-localises nuclear Vav1 to cytosol.* MCF7 cells were transfected with GFP-Vav1 and control siRNA control, BPGAP1 siRNA or re-constitution of HA-BPGAP1 in BPGAP1 siRNA cells. Cells were fixed, permeabilized, stained with anti-BPGAP1 antibody followed by secondary antibody conjugated with Alexa Fluor 555 and Hoechst. Images were acquired using confocal microscopy, scale bar = 30 μm.

Figure S5. The BCH domain of BPGAP1 interacts with Vav1 and is independent of Vav1 activity. *The BCH domain of BPGAP1 interacts with Vav1.* ***(A)*** Schematic diagram of various BPGAP1 truncations: full length (FL), N-terminus with no proline-rich region (NNP) and proline-rich region plus C-terminus (PC). ***(B)*** HEK293T cells co-expressing HA-Vav1 with Flag-tagged full length BPGAP1, PC or NNP were lysed and incubated with anti-Flag beads. Both bound and lysate proteins were analysed with immunoblotting. ***(C)*** *Interaction of BPGAP1 with Vav1 is independent of Vav1 activity*. HEK293T cells transfected with Flag-BPGAP1 and wild-type Vav1, active Vav1-Y174F or inactive Vav1-L278Q mutants were lysed and immunoprecipitated with anti-HA beads. Bound and lysate proteins were analysed by immunoblotting using anti-Flag and anti-HA antibodies. ***(D)*** *Interaction of Vav1 and BCH domain alone (without autoinhibition) does not require EGF stimulation.* HEK293T cells were transfected with Flag-NNP and HA-Vav1 and stimulated with 100 ng/ml EGF for the times indicated. Cells were lysed and immunoprecipitated with anti-Flag beads. Both bound and WCL proteins were analysed by immunoblotting. All graphs in this figure represents mean ± SEM, n ≥3. ** represents *P<0.01*, One Way ANOVA test.

Figure S6. BPGAP1 orchestrates the coupling of RhoA and Rac1 activity. ***(A)*** *Stable expression of BPGAP1 and Vav1 mutants.* Immunoblotting of vector control, wild-type Vav1, RhoGAP inactive mutant-R232A, wild-type BPGAP1, with either the wild-type Vav1 or inactive Vav-L278Q expression in MDA-MB-231 cells. (***B)*** *RacGEF activity of Vav1 is important for the BPGAP1-induced cell motility.* Time-lapse images of cell spreading as described in

Figure 5b. Scale bar: 100 μm. ***(C)*** *Schematic representation of a cell seeded on micropit topographic features.* ***(D)*** Cells were fixed, stained with phalloidin (green) and imaged. Region of interest (ROI; red) was illustrated by Imaris software. Scale bar: 5 μm. ***(E)*** *Representative immunostaining of Vav1 in sections of breast cancer tissue.* Staining of Vav1 was found in the cytoplasmic area of breast cancer epithelial cells with *(i)* negative staining, *(ii)* moderate staining intensity and (iii) strong staining intensity. The mean immunoreactivity score (IRS) of cytoplasmic staining for Vav1 is 100, which is used as the cut off values to stratify Vav1 expression in breast cancer tissues into two groups for univariate analysis. Scale bar: 50μm. ***(F)*** *The cytoplasmic expression level of BPGAP1 positively correlates with Vav1 in breast cancer samples.* The immunoreactivity score (IRS) of Vav1 is plotted against IRS of BPGAP1. r=0.3909, P<0.0001, n=163.

Figure S7. BPGAP1 regulates the dynamic activities of Rac1 and RhoA during cell spreading. ***(A)*** *Rac1 interaction with wild-type BPGAP1 was weaker in the presence of constitutive active RhoA-G14V in MCF7 cells.* MCF7 cells co-expressing the constructs indicated were lysed and immunoprecipitated with anti-HA beads. Bound proteins were detected by the antibodies indicated. The graph represents mean ± SEM of 3 independent experiments; columns of graph are aligned to the lanes of blot, with the conditions indicated. One-way *ANOVA* was used to compare different conditions to the control group. ** represents *P<0.01 and* * represents *P<0.05.* ***(B)*** *Rac1 interaction with BPGAP1 is dependent on the status of RhoA activity governed by the RhoGAP activity of BPGAP1.* HEK293T cells co-expressing

the constructs indicated were lysed and immunoprecipitated with anti-Flag beads. Bound proteins were detected by the antibodies indicated. The graph represents mean ± SEM of 3 independent experiments; columns of graph are aligned to the lanes of blot, with the conditions indicated. One-way *ANOVA* was used to compare different conditions to the control group. ** represents *P<0.01 and* * represents *P<0.05*. ***(C)*** *BPGAP1 association with Vav1 is independent of its RhoGAP a*ctivity during cell spreading. ***(i)*** MCF7 cells were co-transfected with HA-Vav1 and wild type Flag BPGAP1 or RhoGAP mutant BPGAP1-R232A. Transfected cells were seeded on collage-coated plates according to the indicated time, lysed and immunoprecipitated with anti-Flag beads. Both bound and WCL proteins were analysed by immunoblotting. ***(ii)*** The graph represents analysis of relative binding were presented as fold over the cells co-expressing wild-type BPGAP1 and Vav1 in suspension (time = 0). Three independent experiments were performed and represented as mean ± SEM. *n.s* represents not significant, Two-tailed unpaired student's T-test. ***(D)*** *Efficacy of RhoA activator during 30 minutes cell spreading.* MCF7 cells was treated with Rho activator (CN03 1μg/ml) for 2 hours. The cells were seeded for 30 minutes, lysed and incubated with GST-RBD beads. * represents *P<0.05* and Two-tailed unpaired student's T-test was used. **(*E)*** *RacGEF activity of Vav1 is important for the BPGAP1-induced cell motility.* Time-lapse images of cell spreading as described in Figure 6E. Scale bar: 100 μm. Migration tracks (2 representative tracks are illustrated as black and red that were tracked over 2 hours).

Figure S1. BPGAP1 expression is elevated in breast cancer.

A

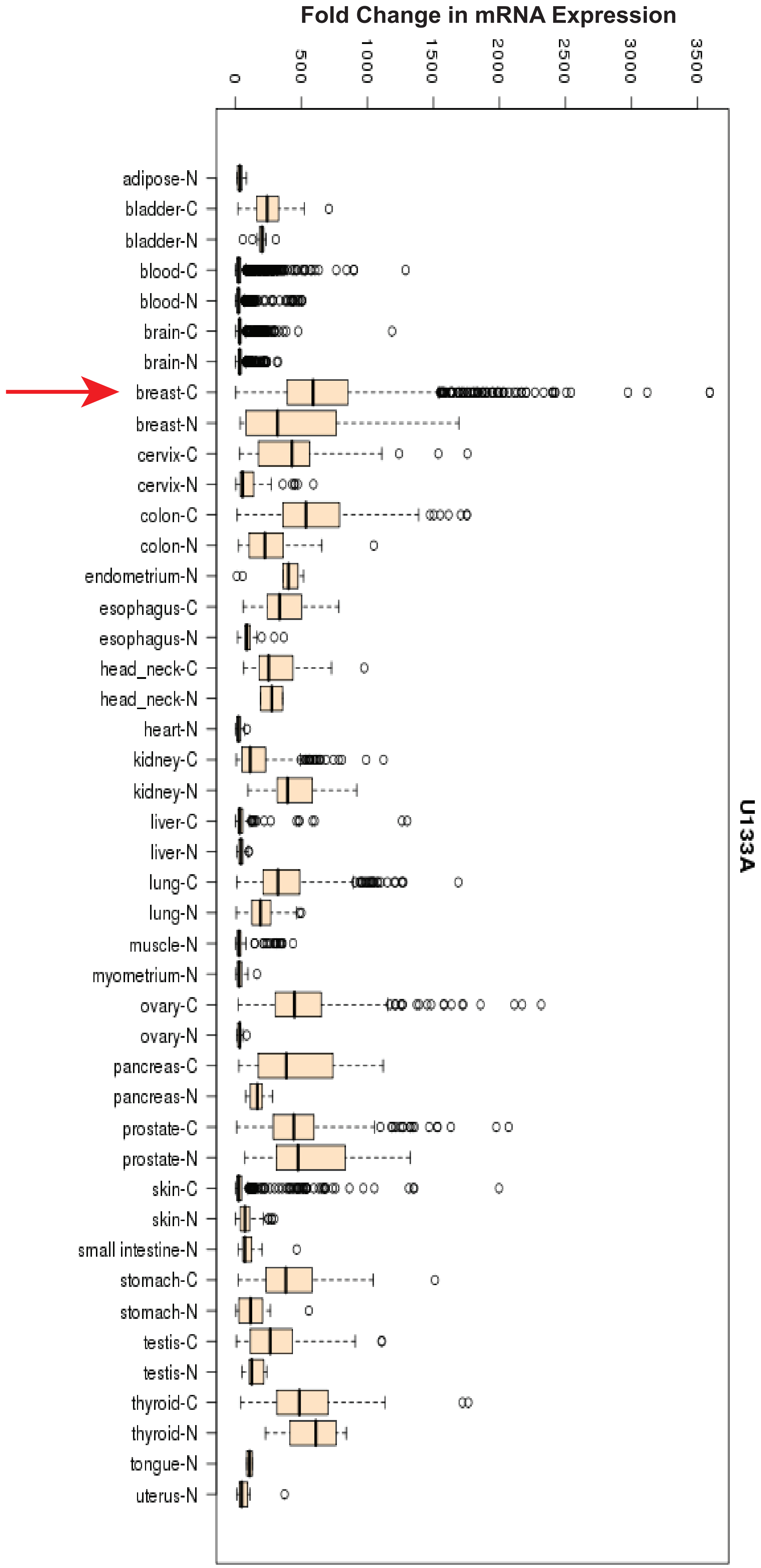

Figure S2. MCF7 cells expresses higher levels of BPGAP1 protein.

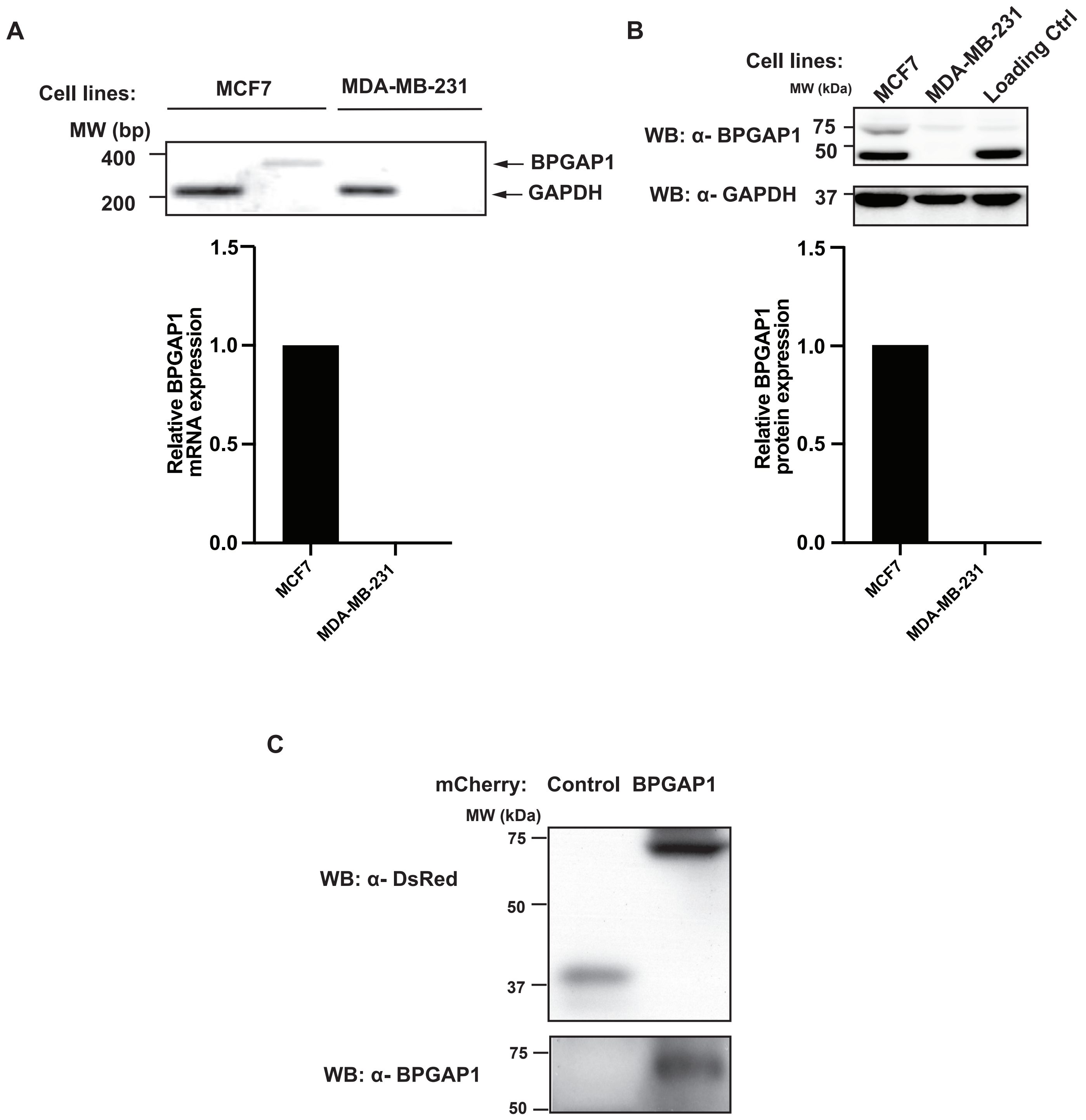

Figure S3. BPGAP1 promotes the motility of MDA-MB-231 breast cancer cells.

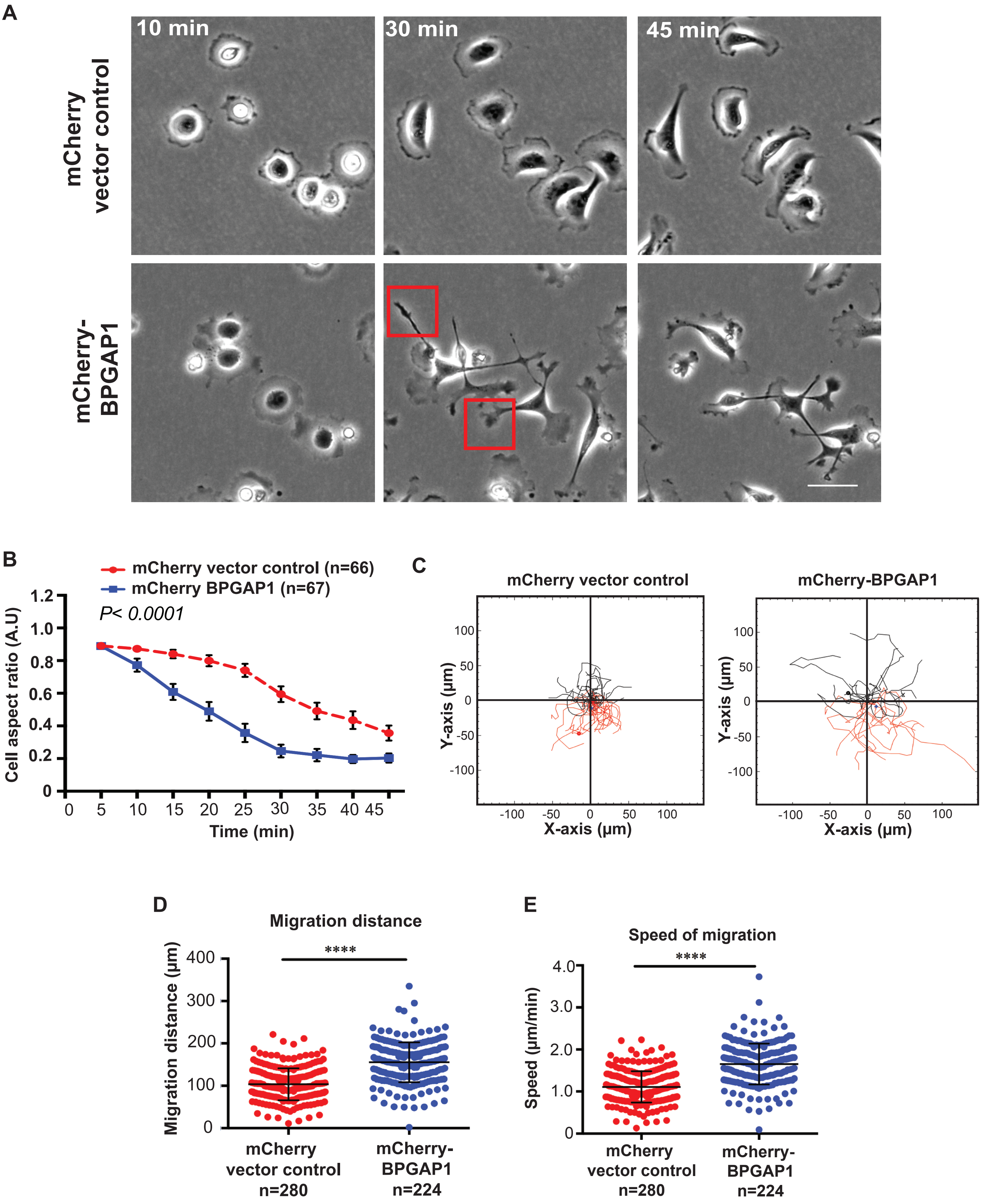

Figure S4. BPGAP1 interacts with Vav and re-localises Vav from nucleus to cytosol and lamellipodia.

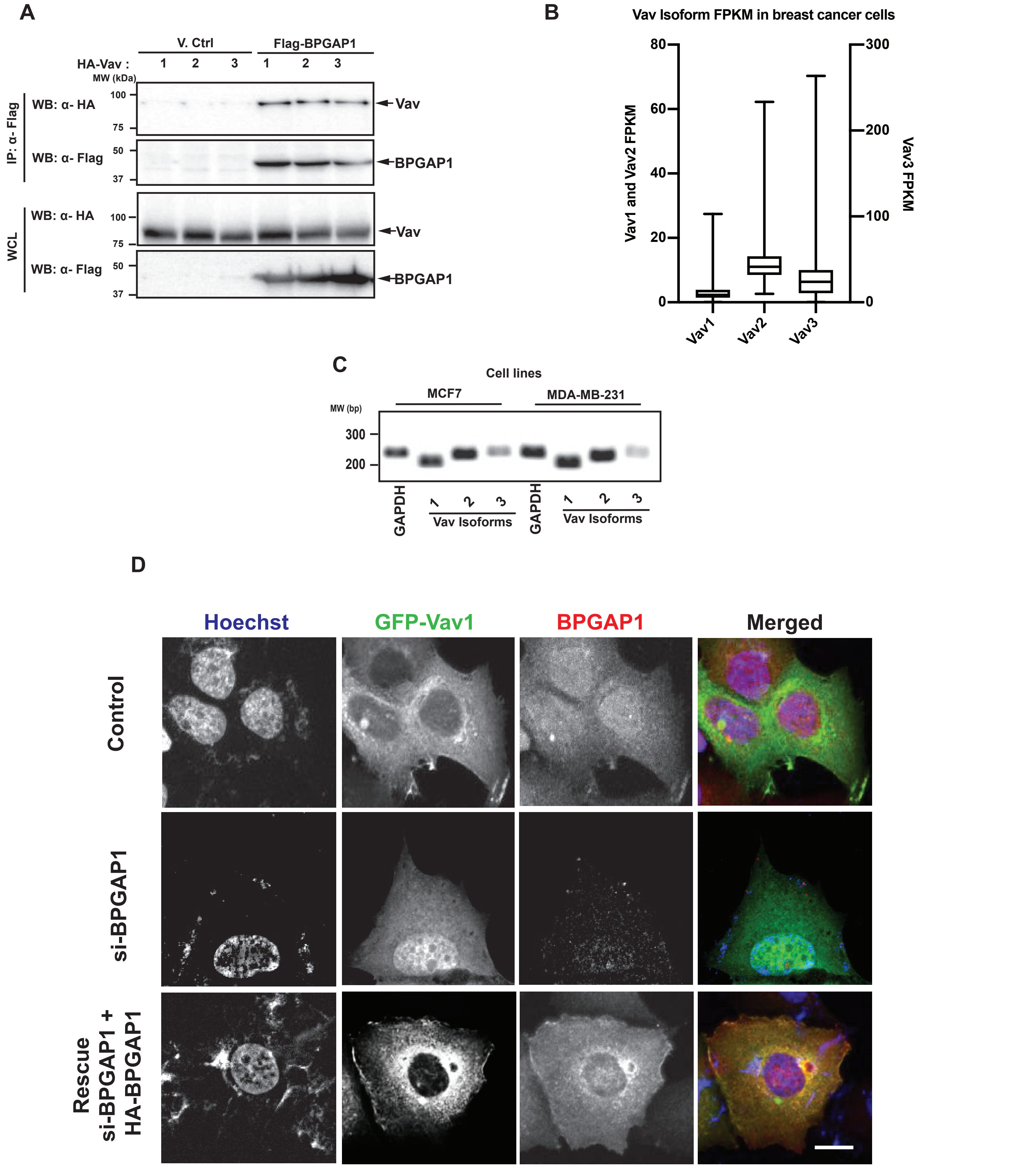

Figure S5. The BCH domain of BPGAP1 interacts with Vav1 and is independent of Vav1 activity.

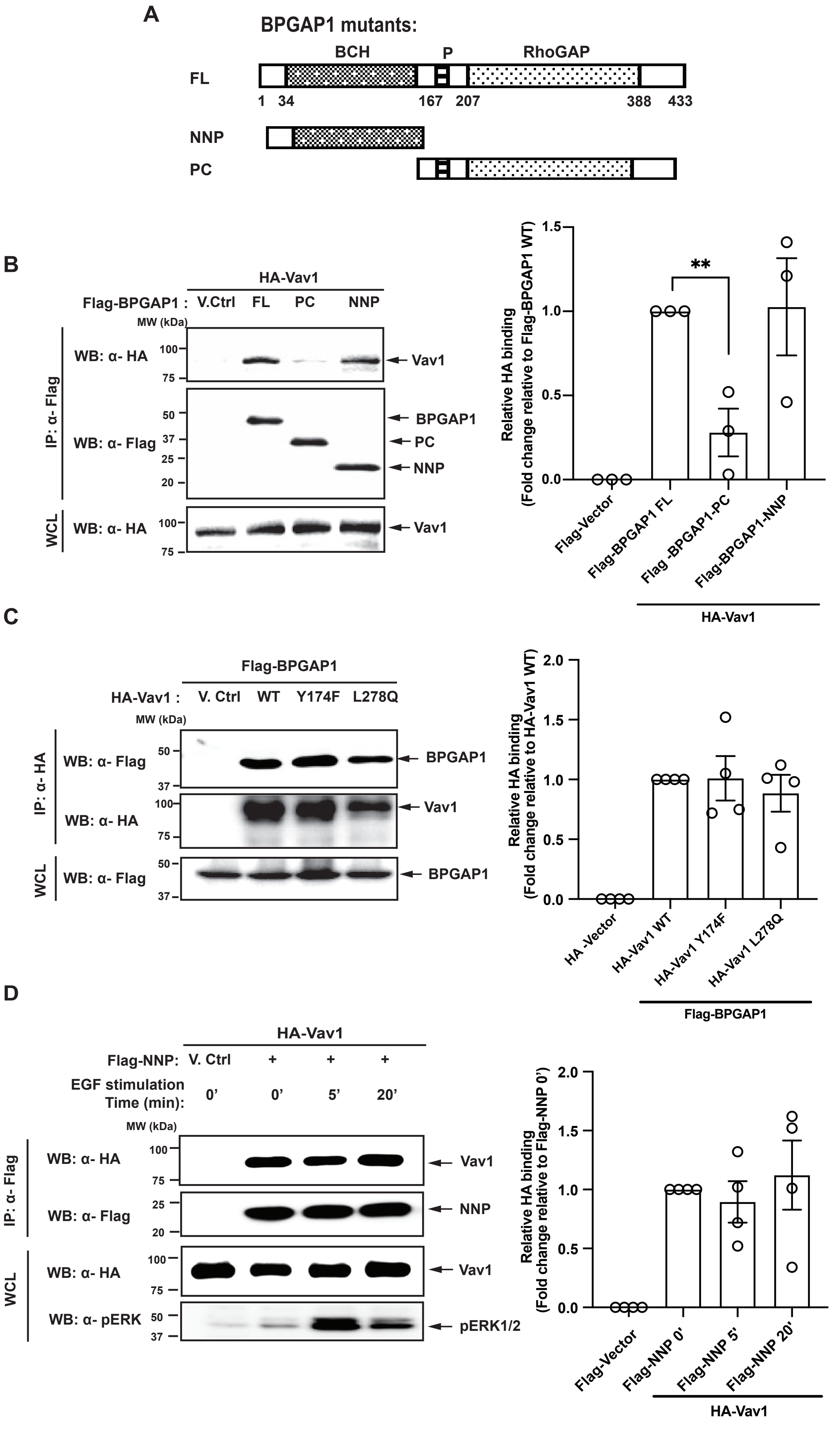

Figure S6. BPGAP1 orchestrates the coupling of RhoA and Rac1 activity.

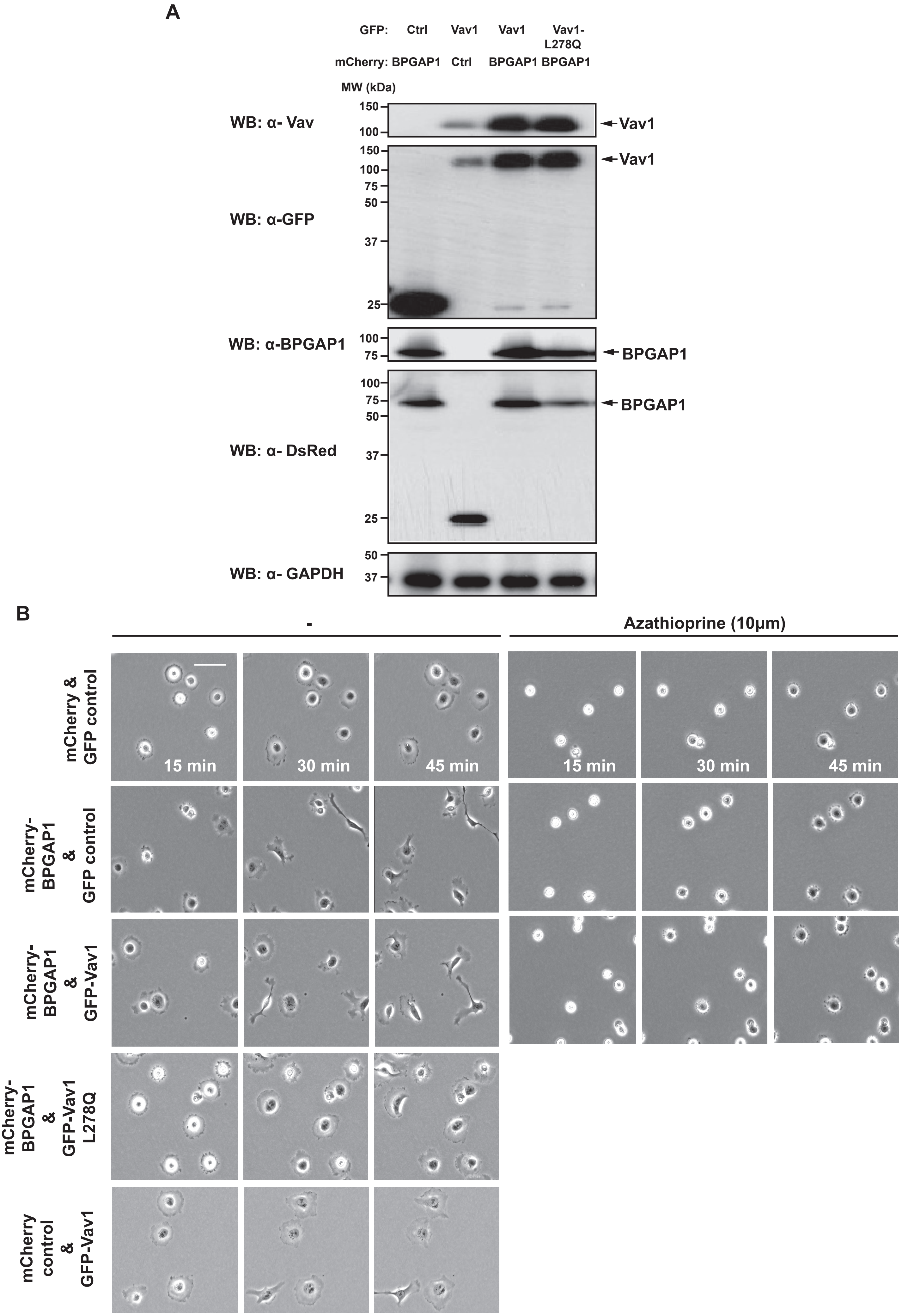

Figure S6. Continue

C

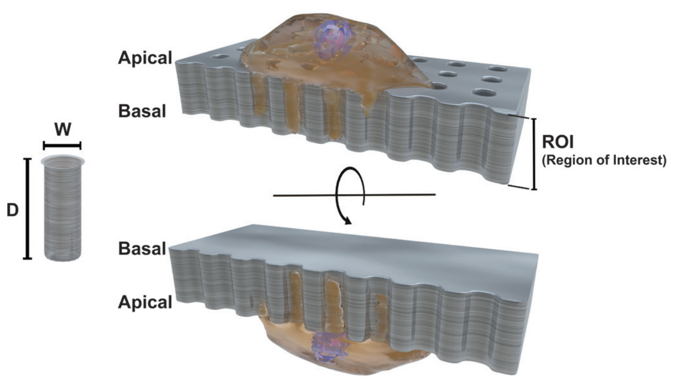

D

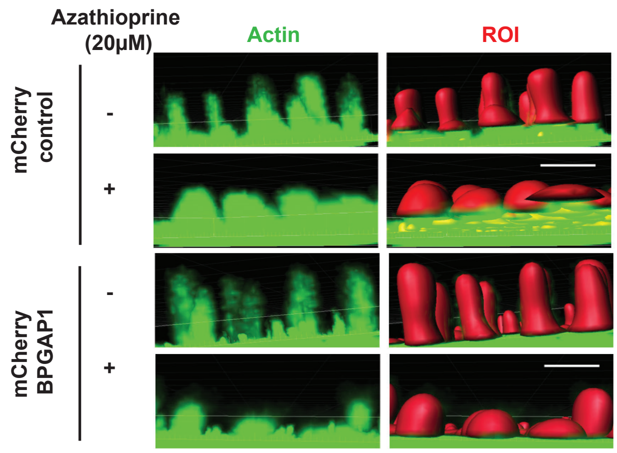

E

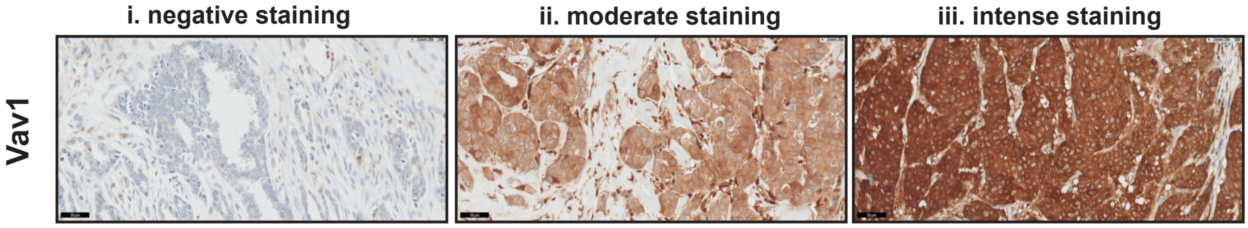

F

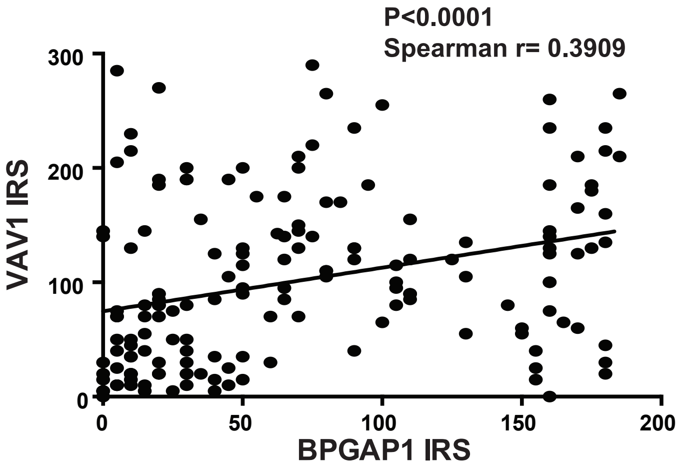

Figure S7.BPGAP1 regulates the dynamic activities of Rac1 and RhoA during cell spreading

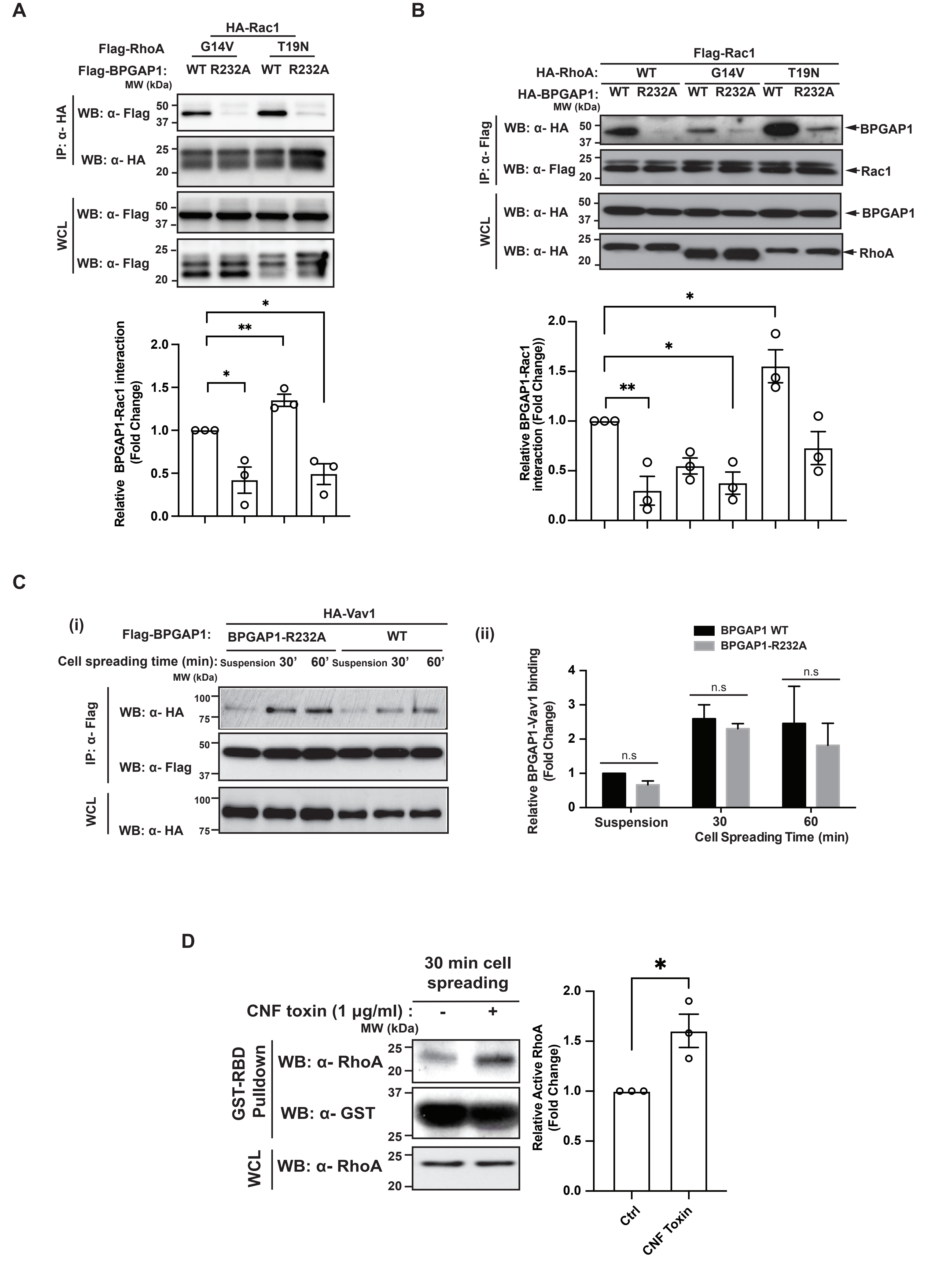

Figure S7. Continue

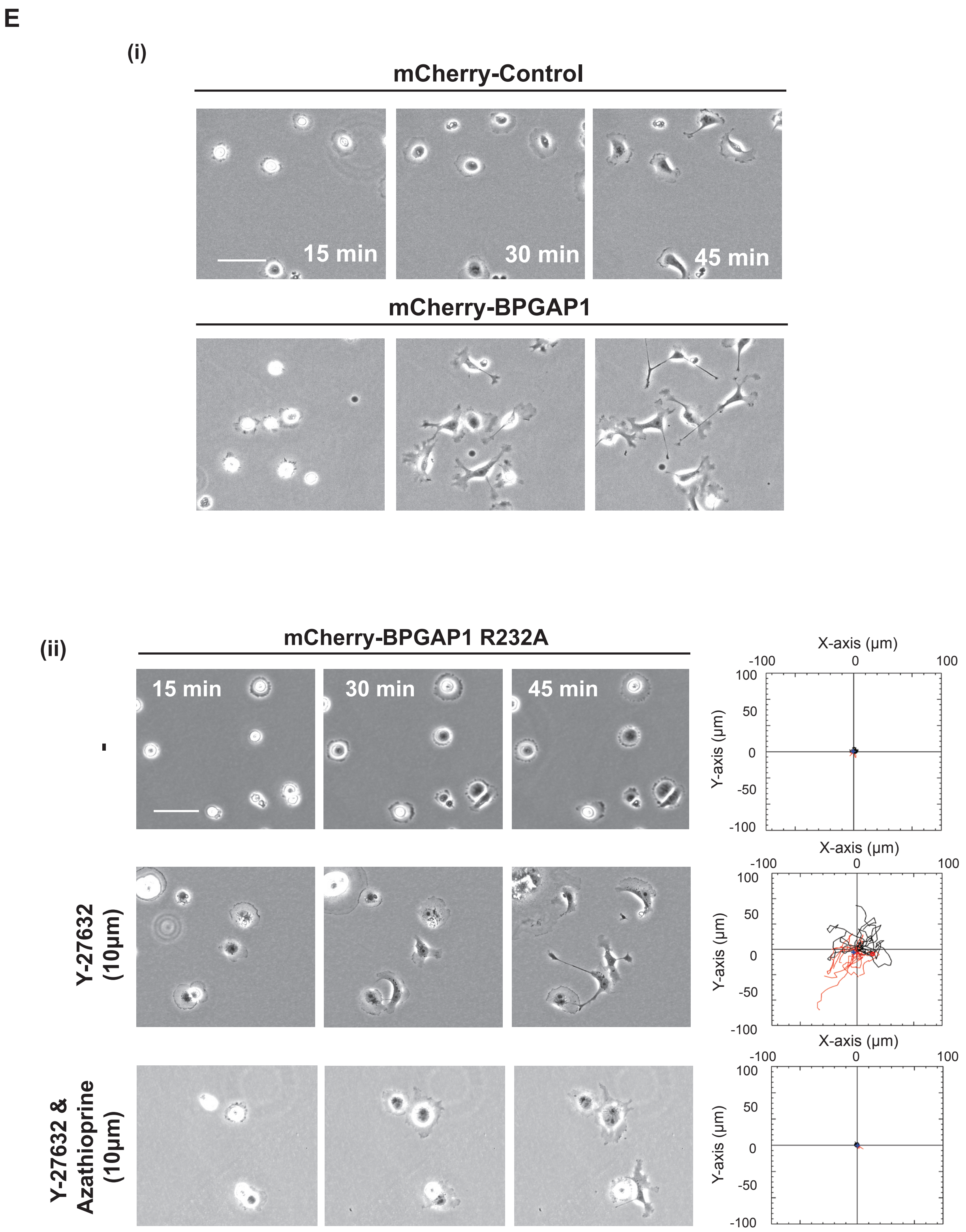

Supplement: Supplementary file 4 [file mbc-34-ar13-s001.pdf]
